# Practical NTRU Signcryption in the Standard Model

**DOI:** 10.3390/e25121651

**Published:** 2023-12-13

**Authors:** Jianhua Yan, Xiuhua Lu, Muzi Li, Licheng Wang, Jingxian Zhou, Wenbin Yao

**Affiliations:** 1 School of Information and Electric Engineering, Ludong University, Yantai 264025, China; 2 School of Cyber Science and Engineering, Qufu Normal University, Qufu 273165, China; 3 Archive Library, Ludong University, Yantai 264025, China; 4 School of Cyberspace Security, Beijing Institute of Technology, Beijing 100081, China; 5 Institute of Science and Technology Innovation, Civil Aviation University of China, Tianjin 300300, China; 6 School of Computer, Beijing University of Posts and Telecommunications, Beijing 100876, China

**Keywords:** lattice, NTRU signcryption, IND-CCA2, partitioning technique, quantum-resistant

## Abstract

Based on the NTRU trapdoor used in NIST’s Falcon, a signcryption scheme following the sign-then-encrypt paradigm is constructed. The existing partitioning technique based on Waters hash over the lattice can not complete the security reduction in the standard model for the signature part due to the “partiality” of the pre-image generated with the NTRU trapdoor. To address this, a variant of Waters hash over small integers is proposed and, the probability of the successful reduction is analyzed. The resulting signcryption achieves existential unforgeability under the adaptive chosen-message attacks. By utilizing the uniqueness of the secret and the noise in an NTRU instance, the tag used in encryption is eliminated. Furthermore, a method to construct tamper-sensitive lattice public key encryption is proposed. This approach implants the ciphertext-sensitive information into the lattice public key encryption and binds it to the encrypted information. The malleability to the public key ciphertext triggers the change of the message–signature pair so that the IND-CCA2 security of the entire ciphertext can be guaranteed by the signature for the message. Thanks to the rational design and the efficiency of the NTRU trapdoor, the computational overhead of the proposed scheme is reduced significantly compared to the existing lattice-based signcryption scheme, reaching orders of magnitude improvement in efficiency. The experiment shows that the proposed scheme is efficient.

## 1. Introduction

Network interaction in complex scenarios provides better services and more convenience for people to live, work, and study. The information security issues in complex scenarios have also become a thorny issue for network workers. In general, a common requirement for information security in complex scenarios is to ensure the comprehensive security of information: confidentiality, integrity, authentication, and non-repudiation. The signcryption due to Zheng [1] can simultaneously guarantee the above-integrated security at a low cost. Designing a secure signcryption scheme has become a research hotspot. Cryptographers have conducted a lot of in-depth research on signcryption based on the number theory and have achieved a series of good results. However, the rapid development of quantum computing [2] has posed a serious threat to the security foundation of traditional cryptography based on number theory. Lattice-based cryptography is becoming the backbone of quantum-resistant cryptography, due to its advantages in efficiency, flexibility, and security in the average case. It has become a common concern to construct a signcryption scheme based on the lattice.

### 1.1. Related Works

To resist quantum attacks, Li et al. proposed the first lattice-based signcryption [3]. After that, the lattice signcryption is developed in three directions: different security models, advanced primitives, and specific applications. Regarding the applications, the signcryption applied to the management of health records has been studied in depth [4,5]. In terms of advanced signcryption primitives, the identity-based signcryption scheme [6], attribute-based signcryption scheme [7] (to support non-interactive fine-grained access control), and multi-recipient scheme [8] were constructed. For the security model, the schemes under the random oracle [9,10,11,12,13], in which [11] is the most efficient due to drawing lessons from the signcryption on Schnorr signature from [14] and the compression technique [15,16], and some schemes under the standard model were constructed.

In 2013, Yan et al. constructed a secure lattice signcryption scheme [17] under the standard model (YWW+13 [17] for short) based on the MP trapdoor [18]. This scheme follows the sign-then-encrypt (StE) paradigm, and the security of the ciphertext must be shifted to a reliance on the unforgeability of the signature with the help of the message authentication code (MAC). In fact, the MAC itself has the ability to improve IND-CCA1 security to IND-CCA2 security. Since the tag used for encryption is generated with the signature value, the lattice-based chameleon hash function is required in the process to vanish the trapdoor for completing the security reduction, which increases the computational overhead and the size of the public key. In 2018, Sato et al. made great progress in proposing a secure signcryption scheme [19] (SS18 [19] for short) under the standard model also based on the MP trapdoor. The lattice-based public key encryption (PKE) is actually an instance of learning with errors (LWE), $c^{\prime }$ = ${\left[A∥u\right]}^{t}s+e$, plus the encoding of the message [0∥μ]⌊q/2⌋. The malleability of the ciphertext lies in the homomorphic computational property of the LWE instance and that of the message. That is, it will affect the decryption, adding ${\left[A∥u\right]}^{t}s^{\prime }$ to the LWE instance, appending a small error $e^{\prime }$ to the LWE instance or superimposing additional information to the message. To eliminate the malleability of the ciphertext, the LWE instance is signed along with the original message in SS18 [19]. Meanwhile, the homomorphic malleability to the plaintext code will of course break the signatures. In this sense, the scheme belongs to the encrypt-then-sign (EtS) paradigm instead of the StE paradigm, as they claim. In 2019, Yang et al. constructed a signcryption scheme [20] (YCL+19 [20] for short) under the standard model based on ring learning with errors (RLWE) [21]. YCL+19 uses the key exchange [22,23] rather than the public key encryption to generate the key for the symmetric encryption, which reduces the size of the public key encryption. The hint information of the lattice-based key exchange is naturally immune to tampering due to its sensitivity to key recovery. However, it incurs a security risk to expose another part of the key exchange. Liu et al. proposed an NTRU-based signcryption [24] (LTTM19 [24] for short) by adopting NTRU-based key encapsulation [25] and an NTRU-based signature [26]. However, the unsigncryption queries can not be implemented in the security reduction under the standard model, so the scheme is not IND-CCA2 secure as they claimed. In the sign-then-encrypt (StE) paradigm, the signature seems more natural than that in encrypt-then-sign (EtS) paradigm since it is signed only for the message. Moreover, the construction of signcryption under the StE paradigm is more concerned with cryptographers [27,28]. It is also a problem of great importance to design a secure lattice-based signcryption scheme following the StE paradigm.

To address the quantum threat to cryptography based on number theory, in 2017, the National Institute of Standards and Technology (NIST) began collecting the post-quantum public key cryptography algorithms through an open, competitive process. The post-quantum cryptographic (PQC) algorithm should meet the following five requirements: secure under the existing computing conditions and quantum computers, fast operation, reasonable communication overhead, can be used as a direct replacement for the existing algorithms and protocols, and broad application scenarios. After many rounds of rigorous screening, NIST announced the screening results of the third round of post-quantum cryptographic algorithm standardization in July 2022. In the four post-quantum algorithms, there are two lattice-based signature algorithms, CRYSTALS-Dilithium [29] and Falcon [30], and they are more efficient than the hash-based signature. In fact, in May 2022, the scientists from Google published the latest research results [31] in the journal “Nature” to illustrate the importance of post-quantum cryptography (PQC) and appeal to transition to PQC. Thus, it is a natural question: ***Can an efficient signcryption scheme following the StE paradigm be designed based on the NIST standard, which is secure in the standard model and does not require MAC transferring?***

### 1.2. Proposed Design

To resist quantum attacks, we construct a signcryption scheme based on NTRU, referred to as SC-NTRU. Our contribution can be summarized in two main points. First, we introduce an approach to improve the security of the encryption segment using the signature segment for the messages in the signcryption. The signature security can be appropriately decreased compared with that in the EtS paradigm. Second, we construct a new abort-resistant hash to adapt to the approximate pre-image scenario, and utilize it to build an NTRU signature secure in the standard model. The reasonable design and efficient trapdoor of SC-NTRU lead to a significant reduction in computational overhead, surpassing existing lattice-based signcryption methods by several orders of magnitude.

We have developed a method to achieve IND-CCA2 security in signcryption by combining three techniques. Firstly, we leverage the uniqueness of the secret and noise used in lattice-based encryption to transform tag-based encryption into general encryption. Secondly, we embed sensitive information related to the ciphertext itself into the ciphertext, binding it to the information to be encrypted using public key encryption (PKE). As the entire encryption is a hybrid encryption, the plaintext hidden in PKE serves as a key for symmetric encryption. Any modification to the ciphertext will consequently modify the key of the symmetric encryption due to their interdependence. Thirdly, we exploit the one-to-one property of symmetric encryption, such that a modified public key ciphertext will produce a modified message–signature pair. Subsequently, the unforgeability of the signature can be utilized to check the malleability and enhance the IND-CCA2 security of the complete ciphertext. It is important to note that the signature here does not need to achieve strong unforgeability while the strong unforgeability for a signature is necessary in the general construction of the IND-CCA2 secure encryption scheme. Since the message–signature pair here is encrypted and concealed from potential adversaries, any attempt to forge a signature would result in a new signature. In summary, the requirement for the signature to enhance encryption to IND-CCA2 security is diminished. Even a strong forgery of the signature supplies no help to unsigncrypting.

A common approach to constructing a secure lattice signature scheme in the standard model is through a partitioning technique based on Waters hash [32]. In [33], this hash function takes the form $H\left(\nu_{0},\nu_{1},\cdots ,\nu_{ℵ}\right)=\sum_{i=0}^{ℵ}{\left(-1\right)}^{\nu_{i}}p_{i}$, with $p_{i}\in Z_{q}$ for i=0,1,⋯,ℵ; it is also referred to as an abort-resistant hash function. The probability of the hash not aborting is demonstrated using the concept of the hyperplane in [34]. However, we find that this hash proposed in [34] can not help us complete the security reduction for the signature component involved in the signcrytion. The pre-image generated by the NTRU trapdoor exhibits a certain level of “partiality”. The entire NTRU trapdoor does not fall into the category of approximate trapdoors, such as that constructed by Chen [35] based on [18], and the pre-image generated by NTRU trapdoor can be exact in its entirety. However, the checkout polynomial only operates on a subset of the pre-image, which is reflected in its form $s_{1}+s_{2}*h_{f}=0$ (refer to Algorithm 2). In other words, the pre-image corresponding to the checkout polynomial is merely an approximate pre-image, as there exists a small error vector $x^{\prime }=y-h_{f}x$. The range of the existing abort-resistant hash is $Z_{q}$. When the hash value operates on the checkout polynomial, the product of the hash value and the short error vector $x^{\prime }$ results in a vector close to the uniform distribution over $Z_{q}^{n}$. Consequently, this product vector makes it impossible to simulate the signature in security reduction. To address this issue, we modify the hash range to a space with a small value. However, this modification leads to a significant increase in the abort probability, which hinders secure reduction. To overcome this issue, we introduce a new random variable that cyclically selects random numbers when the abort condition is met. This helps to avoid premature abandonment. Subsequently, we need to evaluate the probability of successfully completing reduction when an adversary forges a signature. However, this evaluation is not trivial since the hyperplane model for the abort-resistant hash defined over a ring ($Z_{q}$) is inadequate for the hash over small integers.

## 2. Preliminaries

In this paper, the notations are as follows. Z: the set of integers; $Z^{+}$: the set of positive integers; R: the ring R: $=Z\left[x\right]/\left(1+x^{n}\right)$; for a prime *q*, $R_{q}$: =R/q; the bold lowercase letter: polynomial or the vector composed of the coefficients of the polynomial; the bold uppercase letter: matrix; $\tilde{B}$: Gram–Schmidt orthogonalization of the matrix B; ∥x∥: the two-norm of a vector named x; ∥X∥: the maximum of the column vectors, $∥X∥=max_{i}\{∥X_{i}∥\}$.

### 2.1. NTRU Lattice and Hard Problem

**Definition** **1** (NTRU Lattice)**.** *Let R: $=Z\left[x\right]/\left(1+x^{n}\right)$ for some power-of-two integer n. Let $h=g*f^{-1}\;\mathrm{mod}\;q$ for f,g∈R and $q\in Z^{+}$. The corresponding NTRU lattice to h,f is defined as*

$$\begin{array}{c} \begin{array}{c} \Lambda_{h,q}=\left\{\left(u,v\right)\in R^{2}|u+v*h=0\;\mathrm{mod}\;q\right\}. \end{array} \end{array}$$

*$\Lambda_{h,q}$ is a full-rank lattice over $Z^{2n}$ linearly spanned by the row vectors of $A_{h,q}=\left(\begin{array}{cc} -A_{N}\left(h\right) & I_{n}\\ qI_{n} & O_{n} \end{array}\right)$, where $A_{N}\left(h\right)$ denotes the anti-circulant matrix generated by the vector h.*

**Definition** **2** (Decisional Small Polynomial Ratio: DSPR [36])**.** *Let R: $=Z\left[x\right]/\left(1+x^{n}\right)$, $R_{q}=R/q$. For g,f∈R with small coefficients and f invertible over $R_{q}$, the distinguishing problem between the distribution of $h=gf^{-1}\;\mathrm{mod}\;q$ and that of $h^{\prime }\overset{\$}{\leftarrow }R_{q}$ is defined as the decisional small polynomial ratio problem.*

The hardness of the search version of DSPR has been studied in [37].

**Definition** **3** (Search Learning with Errors in a Ring of Integers)**.** *Let *Ψ* be a family of distributions over $K_{R}$ and 2<q∈Z. The RLWE problem RLWE${}_{q,\Psi }$ is to find $s\in R_{q}^{\vee }$ by allowing access to arbitrarily many samples from $A_{s,\Psi }$ for ψ∈Ψ.*

**Proposition** **1** (Hardness of RLWE [21])**.** *Let K denote an arbitrary number field with degree n. Let Z∋q=q(n)≥2 and arbitrary α=α(n)∈(0,1) satisfying $\alpha q\geq \omega (\sqrt{\log n})$. A probabilistic polynomial time quantum reduction can be constructed from K-${\mathrm{DGS}}_{\gamma }$ to $O_{K}$-${\mathrm{LWE}}_{q,\Psi_{\leq \alpha }}$, where $\gamma =\eta_{\epsilon }\left(I\right)\cdot \omega \left(\sqrt{\log n}\right)/\alpha$.*

### 2.2. Trapdoor Generation and Pre-Image Sample Algorithm

**Proposition** **2** (NTRU Key Generation)**.** *Inputting dimension n and modulus q, there is an efficient keyGen algorithm to output public key h and the trapdoor $B=\left(\begin{array}{cc} A_{N}\left(g\right) & -A_{N}\left(f\right)\\ A_{N}\left(g^{\prime }\right) & -A_{N}\left(f^{\prime }\right) \end{array}\right)$, such that h is computationally indistinguishable from the uniform distribution over R, $∥\tilde{B}∥\leq 1.17\sqrt{q}$, and $g,f\leftarrow D_{Z^{n},\eta }$ for $\eta \leq 1.17\sqrt{q/(2n)}$.*

**Proposition** **3** (Pre-Image Sampling [38])**.** *Let $\epsilon =2^{-\lambda }/\left(2n\right)$ for arbitrary $n,\lambda \in Z^{+}$, Δ denote statistical distance. For any basis $B\in Z^{n\times n}$ of $\Lambda_{A,q}$ and arbitrary syndrome vector $u\in Z^{n}$, there is a pre-image sampling algorithm GS, x=GS(B,η,u), such that Ax=umodq and $\Delta \left(D_{\Lambda (B,\eta ,u)},x\right)\leq 2^{-\lambda }$, when $\eta \geq ∥\tilde{B}∥\cdot \zeta_{\epsilon }^{\prime }\left(Z\right)$ and $\zeta_{\epsilon }^{\prime }\left(Z\right)\approx \frac{1}{\pi }\sqrt{\frac{1}{2}ln\left(2+\frac{2}{\epsilon }\right)}$. ϵ can be relaxed to $2^{-\lambda /2}/\left(4\sqrt{2}n\right)$, and $\zeta_{\epsilon }^{\prime }\left(Z\right)\approx \frac{1}{\sqrt{2}\pi }\sqrt{\ln2^{(\lambda +7)/2}n}$.*

**Proposition** **4** (Discrete Gaussian Distribution)**.** *Let *Λ* be an m-dimension lattice. Let real s be the Gauss parameter and $c\in R^{m}$ be the center. The discrete Gauss distribution has the following good properties.*

*1.*  *(Lemma 4.4 of [39]) For any positive real k, $Pr[|x|>ks;x\overset{\$}{\leftarrow }D_{Z,s}]\leq 2e^{-k^{2}/2}$.*
*2.*  *(Lemma 4.4 of [39]) When $s>3/\sqrt{2\pi }$, $D_{Z,s}^{m}\left(x\right)<2^{-m}$ for all vector $x\in Z^{m}$.*
*3.*  *(Lemma 4.4 of [39]) For any positive real k, $Pr[∥x∥>ks\sqrt{m};x\overset{\$}{\leftarrow }D_{Z,s}^{m}]\leq k^{m}e^{(1-k^{2})m/2}$.*
*4.*  *(Lemma 4.3 of [39]) Let $t\in R^{+},\;v\in R^{m}$. Then $Pr\left[\langle z,v\rangle >t\right]\leq 2e^{-t^{2}/(2s^{2}{∥v∥}^{2})}$.*
*5.*  *(Lemma 4.4 of [40]) $\underset{x∼D_{\Lambda ,s,c}}{Pr}[∥x-c∥\geq s\sqrt{m}]\leq \frac{1-\epsilon }{1+\epsilon }2^{-m}$.*
*6.*  *(Lemma 5.2 and Corollary 5.4 of [41]) Let n,m be integers, s real and q prime. When $s\geq \omega (\sqrt{\log m})$, $s\geq \eta_{\epsilon }\left(\Lambda^{⊥}\left(A\right)\right)$ for ϵ∈(0,1/2). When $m\geq 2n\log q,\;s\geq \omega (\sqrt{\log m})$, with $1-2q^{-n}$ probability, the syndrome y=Ax is statistically close to uniform over $Z_{q}^{n}$ for all $A\in Z_{q}^{n\times m}$, $x\overset{\$}{\leftarrow }D_{Z,s}^{m}$.*
*7.*  *(Lemma 2.4 of [42] or lemma 4.4 [40]).*
$$\rho_{s}\left(\Lambda +c\right)\in \left[\frac{1-\epsilon }{1+\epsilon },1\right]\cdot \rho_{s}\left(\Lambda \right).$$

## 3. Signcryption: Syntax and Security Models

In this section, the syntax and security models of signcryption are presented.

### 3.1. Syntax of Signcryption

A signcryption scheme is composed of the following four algorithms:

- **Setup** (λ): This algorithm takes a security parameter λ as input, then returns the public parameter PP.
- **KeyGen** (λ,PP): Input the security parameter λ and the public parameter PP, and output the public key and private key pairs (PK,SK) for users.
- **SignCrypt** $\left(\mu ,PK_{s},SK_{s},PK_{r}\right)$: Take a message μ, the sender’s public key $PK_{s}$ and private key $SK_{s}$, and the recipient’s public key $PK_{r}$, generate a ciphertext *c*.
- **UnSignCrypt** $\left(c,PK_{s},SK_{r}\right)$: Inputting a ciphertext *c*, the sender’s public key $PK_{s}$ and the recipient’s private key $SK_{r}$, this algorithm unsigncrypts the ciphertext and verifies the signature. If the signature can pass the verification, it returns the message μ, otherwise it returns ⊥.

### 3.2. Security Models of Signcryption

To clarify the confidence of the signcryption, an **IND-CCA2** game is introduced (Table 1).

- Initial: The challenger C runs the setup and key generation algorithms to generate public parameters PP, the receiver’s keys $\left(Pk_{r},SK_{r}\right)$, and the sender’s keys $\left(Pk_{s},SK_{s}\right)$, followed by giving $\left(PP,Pk_{r},Pk_{s},SK_{s}\right)$ to an adversary A.
- Phase 1: The adversary A implements the unsigncryption queries in an adaptive manner bounded by polynomial times. If *c* is a valid ciphertext, C replies with the corresponding plaintext μ, otherwise it returns ⊥.
- Challenge: A selects two plaintexts $\mu_{0},\mu_{1}$ with equal length and gives them to C. C tosses a fair coin b∈{0,1} and generates a challenge ciphertext $c^{*}=$ signcrypt$(PK_{s},SK_{s},$$PK_{r},\mu_{b})$ followed by giving $c^{*}$ to A.
- Phase 2: A continues to perform unsigncryption queries as in Phase 1, except for not permitting to query unsigncryption on $c^{*}$.
- Guess: A gives $b^{*}$ as the guess on *b* tossed by C.

Then, the advantage of A to win **Game IND-CCA2** is defined as $Adv\left(A\right)=|\mathrm{Pr}\left[b=b^{\prime }\right]-\frac{1}{2}|$.

**Definition** **4** (Confidentiality of Signcryption)**.** *A signcryption scheme is said to be indistinguishable against inner choose ciphertext attacks (IND-CCA2) if there exists no probabilistic polynomial time inner adversary who can win **Game IND-CCA2** with a non-negligible advantage.*

To capture the unforgeability of the signcryption, an **EUF-CMA game** is introduced (Table 2).

- Initial: The challenger C runs the setup and key generation algorithms to generate public parameters, the keys for the receiver and sender are as in the **IND-CCA2 Game**. Subsequently, C gives $\left(PP,Pk_{s},Pk_{r},SK_{r}\right)$ to A.
- Signcrypt: A chooses messages μ and implements polynomially-bounded signcryption queries by an adaptive approach. C replies with the corresponding ciphertexts *c*.
- Forge: A outputs $c^{*}$, which contains a new signature for some μ that has not been previously queried.

Then, the advantage of A to win **Game EUF-CMA** is defined as Adv(A)=Pr[(μ,σ) = Unsigncrypt$\left(c^{*})\;\;\mathrm{and}\;\;N\right]$, where N denotes the fact that σ is a valid signature for μ not discoved by the unsigncryption queries.

**Definition** **5** (Existential Unforgeability of Signcryption)**.** *A signcryption scheme is said to be existentially unforgeable against inner chosen message attacks (EUF-CMA) if no probabilistic polynomial time forgery can win **Game UF-CMA** with a non-negligible advantage.*

## 4. Signcryption Based on NTRU

In this section, a signcryption scheme based on NTRU is proposed, followed by its correctness and parameter settings.

### 4.1. Construction

The symmetric encryption ENC involved in the proposed scheme is IND-OT secure and one-to-one. That is, for a plaintext μ and symmetric key *k*, there is only one ciphertext *c* satisfying DEC${}_{k}\left(c\right)=\mu$.

- Setup$\left(1^{\lambda }\right)$: On inputting a security parameter $1^{\lambda }$, generate the public parameters and hash functions.
  1. Choose hash functions: $H_{0}:{\left\{0,1\right\}}^{*}\to {\left\{0,1\right\}}^{ℵ}$, $H_{1}:R_{q}\to R_{q}$, $H_{2}:R_{q}\times R_{q}\to {\left\{0,1,2,3\right\}}^{n}$.
  2. $y,u\overset{\$}{\leftarrow }R_{q}$.
  3. $z_{i}\overset{\$}{\leftarrow }R_{q}$ for i=0,1,⋯,ℵ.
  4. publish public parameters $\left(y,u,{\left\{z_{i}\right\}}_{i=1}^{ℵ}\right)$.
- KeyGen$\left(1^{\ell }\right)$: User *i* generates it own public key and private key.
  1. (B,h)←KeyGen(n,q) to satisfy $B\left(\begin{array}{c} i\\ h \end{array}\right)=0$ where $B=\left(\begin{array}{cc} A(g) & -A(f)\\ A(G) & -A(F) \end{array}\right)\in Z^{2n\times 2n}$, The coefficient vector of i is ${\left(1,0,0,\cdots ,0\right)}^{t}$.
  2. $b,d\overset{\$}{\leftarrow }R_{q}$.
  3. Publish (b,d,h) as public key and keep B as private key. Namely, the sender’s (resp. receiver’s) public and private key are $\left(\left(b_{s},d_{s},h_{s}\right),B_{s}\right)$ (resp. $\left(\left(b_{r},d_{r},h_{r}\right),B_{r}\right)$).
- SignCrypt$\left(\mu ,h_{r},B_{s},{\left\{z_{i}\right\}}_{i=0}^{ℵ}\right)$.
  1. $k\overset{\$}{\leftarrow }{\left\{0,1,2,3\right\}}^{n}$.
  2. ν: $=H_{0}\left(\mu ,b_{r},k\right)$.
  3. z: $=z_{0}+\sum_{i=1}^{ℵ}{\left(-1\right)}^{\nu_{i}}z_{i}$.
  4. $x_{1}\leftarrow D_{Z^{n},\eta_{1}}$.
  5. t: $=y-zx_{1}$.
  6. $\left(x_{0}^{\prime },x_{0}\right)$: $=\left(t,0\right)-\mathrm{SamplePre}(B_{s},\eta_{1},\left(t,0\right))$.
  7. x: $={\left(x_{0},x_{1}\right)}^{t}$.
  8. $r\overset{\$}{\leftarrow }{\left\{-1,0,1\right\}}^{n}$, $e_{0},e_{2}\overset{\$}{\leftarrow }{\left\{-\varrho ,\cdots ,\varrho \right\}}^{n}$, $e_{1}\overset{\$}{\leftarrow }{\left(\left[-\varrho \sqrt{n},\varrho \sqrt{n}\right]\cap Z\right)}^{n}$.
  9. $c_{0}$: $=rh_{r}+e_{0}$.
  10. $c_{1}$: $=r\left(b_{r}+H_{1}\left(c_{0}\right)d_{r}\right)+e_{1}$.
  11. $c_{2}^{\prime }$: $=\lfloor \left(ru+e_{2}\right)/2^{\ell }\rfloor$.
  12. $c_{2}$: $=c_{2}^{\prime }+\left\lfloor \left(\left(H_{2}\left(c_{1},c_{2}^{\prime }\right)⊕k\right)\left\lfloor q/8\right\rfloor \right)/2^{\ell }\right\rfloor$.
  13. $c_{3}$: $={\mathrm{Enc}}_{H_{3}\left(k\right)}\left(x,\mu \right)$.
  14. output $\left(c_{0},c_{1},c_{2},c_{3}\right)$.
- UnSignCrypt$\left(c_{0},c_{1},c_{2},c_{3},B_{r},h_{s},{\left\{z_{i}\right\}}_{i=0}^{ℵ}\right)$.
  1. $s_{1}\leftarrow D_{Z^{n},\eta_{2}}$.
  2. t: $=u-s_{1}\left(b_{r}+H_{1}\left(c_{0}\right)d_{r}\right)$.
  3. $\left(s_{0}^{\prime },s_{0}\right)=\left(t,0\right)-\mathrm{SamplePre}\left(B_{r},\eta_{2},\left(t,0\right)\right)$.
  4. w: $=c_{2}*2^{\ell }-s_{1}c_{1}-s_{0}c_{0}$.
  5. $\tilde{k}:=\left\lfloor \frac{w}{\left\lfloor q/8\right\rfloor }\right\rceil$.
  6. $c_{2}^{\prime }=c_{2}-\left\lfloor \tilde{k}\left\lfloor q/8\right\rfloor /2^{\ell }\right\rfloor$.
  7. $k=\tilde{k}⊕H_{2}\left(c_{1},c_{2}^{\prime }\right)$.
  8. (x,μ): $={\mathrm{Dec}}_{H_{3}\left(k\right)}\left(c_{3}\right)$.
  9. ν: $=H_{0}\left(\mu ,b_{r},k\right)$.
  10. z: $=z_{0}+\sum_{i=1}^{ℵ}{\left(-1\right)}^{\nu_{i}}z_{i}$.
  11. If $∥\left(h_{s},z\right)x-y∥\;\leq \frac{1}{2\pi^{3}}ℵn^{5/2}{\left(\ln\left(2^{(7+\lambda )/2}n\right)\right)}^{3/2}$ return μ, otherwise return ⊥.

### 4.2. Correctness

In the proposed scheme, we draw lessons from [43] to use the ciphertext generated in the previous step (i.e., $c_{0}$) as the tag to produce the subsequent ciphertext. On the one hand, it can transform a tag encryption to a normal encryption. On the other hand, when $c_{0}$ is determined, the rest of the ciphertexts are relatively determined except for the influence of errors and the value to be encrypted, which is important for the security reduction (reference to Theorem 1). We give the unique witness for NTRU as follows.

**Lemma** **1** (Unique Witness)**.** *Let $\varrho =20\log n,\;\;q=\frac{5}{\pi^{3}}\varrho ℵn^{3}{\left(\ln\left(2^{(7+\lambda )/2}n\right)\right)}^{3/2}$ for all but a negligible fraction of h∈R and any syndrome c∈R be the number of the pair $\left(r,e\right)\in {\left\{-1,0,1\right\}}^{n}\times {\left\{-\varrho ,\cdots ,\varrho \right\}}^{n}$ to satisfy that c=rh+e is no more than one.*

**Proof.**  For each c∈R, suppose that there exist two different $\left(r,e\right)\neq \left(r^{\prime },e^{\prime }\right)\in {\left\{-1,0,1\right\}}^{n}\times {\left\{-1,0,1\right\}}^{n}$ satisfying $r+he=r^{\prime }+he^{\prime }=c$. Then, $\left(r-r^{\prime }\right)h=e^{\prime }-e\in {\left\{-2\varrho ,\cdots ,2\varrho \right\}}^{n}$. To complete this lemma, it only needs to argue that $∥\tilde{r}{h∥}_{\infty }>4\varrho +1$ with overwhelming probability, for $\tilde{r}\in {\left\{-2,\cdots ,2\right\}}^{n}$. Let *£* denote the *n*-dimension open $\ell_{\infty }$ cube with the radius of each dimension being 4ϱ+1 (edge length 8ϱ+2). Then, for any fix $\tilde{r}\in {\left\{-2,\cdots ,2\right\}}^{n}$, the probability that $\tilde{r}h$ falls into some cubic *£* is ${\left(8\varrho +2\right)}^{n}/q^{n}$. According to the union bound, for all $\tilde{r}\in {\left\{-2,\cdots ,2\right\}}^{n}$, the probability of $\tilde{r}h$ falling into some *£* is no more than

$$5^{n}{\left(\frac{8\varrho +2}{q}\right)}^{n}={\left(\frac{40\varrho +10}{q}\right)}^{n}<{\left(\frac{41\varrho }{\frac{5}{\pi^{3}}\varrho ℵn^{3}{\left(\ln\left(2^{(7+\lambda )/2}n\right)\right)}^{3/2}}\right)}^{n}={\left(\frac{41\pi^{3}}{5ℵn^{3}{\left(\ln\left(2^{(7+\lambda )/2}n\right)\right)}^{3/2}}\right)}^{n},$$

$$5^{n}{\left(\frac{8\varrho +2}{q}\right)}^{n}={\left(\frac{40\varrho +10}{q}\right)}^{n}<{\left(\frac{41\varrho }{\frac{6\sqrt{2}}{\pi^{3}}\varrho ℵn^{3}{\left(\ln\left(2^{(7+\lambda )/2}n\right)\right)}^{3/2}}\right)}^{n}={\left(\frac{41\pi^{3}}{6\sqrt{2}ℵn^{3}{\left(\ln\left(2^{(7+\lambda )/2}n\right)\right)}^{3/2}}\right)}^{n},$$

which is negligible. That is, for all but a negligible fraction of h, $∥\tilde{r}{h∥}_{\infty }>4\varrho +1$.    □

By the way, in the scheme, the converted ciphertext with each dimensional error less than $\frac{4}{\sqrt{2}\pi^{2}}\varrho n^{3}{\left(\ln\left(2^{(7+\lambda )/2}n\right)\right)}^{3/2}$, the unique witness also holds. The corresponding probability is ${\left(\frac{8}{\sqrt{2}\pi^{2}}\varrho n^{3}{\left(\ln\left(2^{(7+\lambda )/2}n\right)\right)}^{3/2}/q\right)}^{n}5^{n}\approx {\left(5/36\right)}^{n}$, which is negligible.

In order to guarantee the correctness and security of the proposed scheme, the following requirements should be satisfied.

- The RLWE should be hard $\alpha q\geq \omega (\sqrt{\log n})$ (Proposition 1).
- The pre-image sample algorithm works well, $\eta \;\geq \;∥\tilde{B}∥\cdot \zeta_{\epsilon }^{\prime }\left(Z\right)$ for $\zeta_{\epsilon }^{\prime }\left(Z\right)\approx \frac{1}{\pi }$$\sqrt{\frac{1}{2}ln\left(2+\frac{2}{\epsilon }\right)}$ and $\epsilon =2^{-\lambda /2}/\left(4\sqrt{2}n\right)$ [38], where B is the corresponding basis of a lattice (Proposition 3).
- In the security reduction, a simulated trapdoor (Algorithm 1) can be used for sampling (Proposition 3).
- The correctness of the unsigncryption requires that both the error in the public key encryption and the error in the signature are small enough to guarantee security.

Therefore, the Gaussian parameter and the modulus can be set as follows. $\eta_{2}$: Gaussian parameter for decryption; $\eta_{1}$: Gaussian parameter for signature. The correctness of the proposed scheme is implied in Lemmas 11 and 16.

(1) $$\begin{array}{c} \begin{array}{cc} \; & \eta_{1}=\frac{1}{2\pi^{3}}ℵn^{2}{\left(\ln\left(2^{(7+\lambda )/2}n\right)\right)}^{3/2},\;\;\;\;\;\eta_{2}=\frac{1}{\sqrt{2}\pi^{2}}n{\left(\ln\left(2^{(7+\lambda )/2}n\right)\right)}^{3/2},\;\;\;\;\varrho =10{\left(\log n\right)}^{3/4},\\ & q=\frac{6\sqrt{2}}{\pi^{3}}\varrho ℵn^{3}{\left(\ln\left(2^{(7+\lambda )/2}n\right)\right)}^{3/2},\;\;\;\;\;\eta_{3}=\frac{1}{\sqrt{2}\pi^{2}}\sqrt{n}\ln\left(2^{(7+\lambda )/2}n\right) \end{array} \end{array}$$

## 5. Security and Performance

The confidentiality and unforgeability of SC-NTRU are demonstrated in Section 5.1. The efficiency of the SC-NTRU is evaluated by analyzing the numbers of different kinds of computations in Section 5.2. An experiment for SC-NTRU is presented in Section 5.3.

### 5.1. Security

The IND-CCA2 security of the proposed scheme is based on a variant of the search RLWE implied in [38]. We formalize it as follows.

**Definition** **6** (Variant of Search RLWE)**.** *Let ϱ,t be small integers, $s\in R_{q}$ with ${∥s∥}_{\infty }\leq \varrho$. Let ℓ denote a positive integer, and *Ψ* be a family of distributions over $K_{R}$. For i∈[ℓ+1], $e_{i}\leftarrow \Psi$, $a_{i}\leftarrow R_{q}$. The search RLWE is to find $k\in {\left[2^{t-1}\right]}^{n}$ from $c_{i}=a_{i}s+e_{i}$ for i∈[ℓ] and $c_{\ell +1}=a_{\ell +1}s+e_{\ell +1}+k\left\lfloor q/2^{t}\right\rfloor$.*

**Remark** **1.** *The variant is as hard as the standard search LWE. Suppose there exists an adversary A who can find the correct k. Then, A can compute $c_{\ell +1}^{\prime }=a_{\ell +1}s+e_{\ell +1}$ due to $k\lfloor q/2^{t}\rfloor$ hidden by $c_{\ell +1}^{\prime }$. That is, A has the ability to compute $c_{\ell +1}^{\prime }$ from ${\left\{c_{i}\right\}}_{i=1}^{\ell }$. One method is that A learns r from ${\left\{c_{i}\right\}}_{i=1}^{\ell }$, then A uses the same r to get k from $c_{\ell +1}$. This means that the variant of the search RLWE can be reduced to the standard search RLWE in polynomial time. The other method is that A can find the mapping from ${\left\{a_{i}\right\}}_{i=1}^{\ell }$ to $a_{\ell +1}$. The mapping must have the form as $a_{\ell +1}=\sum_{i=1}^{\ell }x_{i}a_{i}+y$, where $x_{i},y$ are sampled from $R_{q}$ with small coefficients. Otherwise, $a_{\ell +1}=\sum_{i=1}^{\ell }x_{i}a_{i}^{\kappa_{i}}+y$, $1\neq \kappa_{i}\in Z$. When the mapping is applied to ${\left\{c_{i}\right\}}_{i=1}^{\ell }$, the error will increase almost to the uniform distribution, which leads to failing to obtain k from $c_{\ell +1}$. In fact, it is also a search RLWE problem to find the mapping $a_{\ell +1}=\sum_{i=1}^{\ell }x_{i}a_{i}^{\kappa_{i}}+y$.*

**Theorem** **1** (IND-CCA2). *Under the parameter settings as in Equation (1), the proposed signcryption scheme is IND-CCA2 secure against inner adversaries in the standard model, as long as the RLWE${}_{n,m,q}$ problem and DSPR problem are computationally intractable.*

**Proof.**  The theorem can be proven by a series of games G${}_{0}$, G${}_{1}$, ⋯, G${}_{13}$. In each game, the adversary A’s probability of success is $Pr[A_{i}]$ for i∈{0,1,⋯,13}. G${}_{0}$ is the real IND-CCA2 security game. In G${}_{0}\cdots$ G${}_{10}$, the challenge ciphertexts are all hybrid encryption ciphertexts. It is proven that the successive games satisfy the indistinguishability or the game transitions based on failure events to A. Thus, the difference between attacking in G${}_{0}$ and attacking in G${}_{10}$ is guaranteed to be negligible. Due to the security of the symmetric encryption involved, the ciphertext of the symmetric encryption $c_{3}$ does not reveal any information about the plaintext and the corresponding signature.

Only using symmetric encryption, a direct attack on the message–signature pair is no less difficult than an attack on the symmetric key. According to the unforgeability of the signature and the security of the symmetric encryption, it is proven that it is impossible to manipulate $c_{3}$ to obtain a valid ciphertext to help the attack. Therefore, ignoring the ciphertext $c_{3}$ in the challenge ciphertext of G${}_{11}$, the transformation does not increase the hardness of the adversary’s attack. Following that, it is shown that the game transitions based on failure events are satisfied from game G${}_{11}$ to G${}_{13}$. In G${}_{13}$, the challenge ciphertext is an RLWE instance, and the probability that the adversary succeeds to obtain the information encrypted by the public key is negligible. Thus, the A’s success probability to attack in G${}_{0}$ is also negligible. This means that the proposed scheme is IND-CCA2 secure. The games are as follows.

- G${}_{0}$: The game G${}_{0}$ is the original IND-CCA2 game, namely, $h_{r}\leftarrow \mathrm{KeyGen},b_{r},d_{r}\leftarrow Z_{q}^{n}$.
  – Setup: Choose hash functions: $H_{0}:{\left\{0,1\right\}}^{*}\to {\left\{0,1\right\}}^{ℵ}$, $H_{1}:R_{q}\to R_{q}$, $H_{2}:R_{q}\times R_{q}\to {\left\{0,1,2,3\right\}}^{*}$, and public parameters $y,u\overset{\$}{\leftarrow }R_{q}$.
  – KeyGen: Generate the public and private keys: $\left(B_{s},h_{s}\right)\leftarrow \mathrm{KeyGen}\left(n,q\right)$, $\left(B_{r},h_{r}\right)$←KeyGen(n,q), $b_{s},d_{s}\overset{\$}{\leftarrow }R_{q},z_{i}\overset{\$}{\leftarrow }R_{q}$ for i=0,1,⋯,ℵ. Publish $\left(b_{s},d_{s},h_{s},h_{r}\right)$ as the public key and keep $B_{s}$ and $B_{r}$ as private keys.
  – Phase I: Upon receiving an unsigncryption query from A, C uses the private key $B_{r}$ to unsigncrypt as in the proposed scheme. If the signature satisfies the constraint $∥\left(h_{s},z\right)x-y∥\;\leq \;\frac{1}{2\pi^{3}}ℵn^{5/2}{\left(\ln\left(2^{(7+\lambda )/2}n\right)\right)}^{3/2}$, C returns the message μ, otherwise it returns ⊥.
  – Challenge: After a polynomial round of interaction with C, A gives a satisfied signal to C. C randomly chooses a message μ and generates the challenge ciphertext $c^{*}=\left(c_{0}^{*},c_{1}^{*},c_{2}^{*},c_{3}^{*}\right)$ according to the steps of sincryption in the proposed scheme, then gives $c^{*}$ to A.
  – Phase II: If the ciphertext for unsigncryption from A is $c^{*}$, C replies with ⊥ directly. Otherwise, C unsigncrypts the querying ciphertext as in Phase I.
- G${}_{1}$: In the game G${}_{1}$, only the producing method of $b_{r}$ is changed. Let $b_{r}=h_{r}p+e$ where $p\leftarrow {\left\{-1,0,1\right\}}^{n},e\leftarrow {\left\{-\varrho ,\cdots ,\varrho \right\}}^{n}$. Since C has the normal private key, the unsigncryption approach is the same as in G${}_{0}$.
- G${}_{2}$: Before publishing the public keys, C generates a challenge ciphertext $c^{*}=\left(c_{0}^{*},c_{1}^{*},c_{2}^{*},c_{3}^{*}\right)$ normally, namely $c_{0}^{*}=rh_{r}+e_{0}$, $c_{1}^{*}=r\left(b_{r}+H_{1}\left(c_{0}^{*}\right)d_{r}\right)+e_{1}$, $c_{2}^{\prime }$: $=\lfloor \left(ru+e_{2}\right)/2^{\ell }\rfloor$, $c_{2}^{*}$: $=c_{2}^{\prime }+\left\lfloor \left(\left(H_{2}\left(c_{1}^{*},c_{2}^{\prime }\right)⊕k\right)\left\lfloor q/8\right\rfloor \right)/2^{\ell }\right\rfloor$, $c_{3}^{*}$: $={\mathrm{Enc}}_{H_{3}\left(k\right)}\left(x,\mu \right)$ for $r\overset{\$}{\leftarrow }{\left\{-1,0,1\right\}}^{n},e_{0},e_{2}\overset{\$}{\leftarrow }{\left\{-\varrho ,\cdots ,\varrho \right\}}^{n},e_{1}\overset{\$}{\leftarrow }{\left(\left[-\varrho \sqrt{n},\varrho \sqrt{n}\right]⋂Z\right)}^{n}$.
- G${}_{3}$: In the game G${}_{3}$, C continues changing $b_{r}$ as $b_{r}=h_{r}p+e-H_{0}\left(c_{0}^{*}\right)d_{r}$ where the method to generate p,e is identical to that in G${}_{2}$.
- G${}_{4}$: The game G${}_{4}$ is the same as G${}_{3}$, except for the approach to produce $d_{r}$. C generates $d_{r}$ by KeyGen algorithm, $d_{r}\leftarrow \mathrm{KeyGen}$, instead of $d_{r}\overset{\$}{\leftarrow }R_{q}$.
- G${}_{5}$: C answers with ⊥ to the unsigncryption queries with the kind of ciphertext $\left(c_{0}^{*},c_{1},c_{2},c_{3}\right)$ in phase I. The others remain the same as in the game G${}_{5}$.
- G${}_{6}$: In this game, in phase I, C answers all the unsigncryption queries normally, but C replies with ⊥ to the queries with ciphertext $\left(c_{0},c_{1},c_{2}\right)$ satisfying $c_{0}\neq c_{0}^{*}$ but $H_{1}\left(c_{0}\right)=H_{1}\left(c_{0}^{*}\right)$.
- G${}_{7}$: In phase II, C answers with ⊥ to unsigncryption queries with the kind of ciphertext $\left(c_{0}^{*},c_{1}^{*},c_{2}^{*},c_{3}\right)$ meeting $c_{3}\neq c_{3}^{*}$. Except for the cases above, C responds to the unsigncryption queries as in G${}_{6}$.
- G${}_{8}$: In phase II, C responds with ⊥ to the unsigncryption queries with the form $\left(c_{0}^{*},c_{1},c_{2},c_{3}\right)$, satisfying $\left(c_{1},c_{2}\right)\neq \left(c_{1}^{*},c_{2}^{*}\right)$. Replying to the other unsigncryption queries keeps the same as in G${}_{7}$.
- G${}_{9}$: In this game, C produces $h_{r}\leftarrow Z_{q}^{n}$ instead of $h_{r}\leftarrow \mathrm{KeyGen}$. Moreover, C will use $d_{r}$ to answer the unsigncryption queries. The others are identical to the game G${}_{8}$.
- G${}_{10}$: In this game, C produces the challenge ciphertext by collaborating with a signer, which for convenience will be called signature oracle $O_{sign}$. First, C generates $\left(c_{0}^{*},c_{1}^{*},c_{2}^{*}\prime \right)$ normally as in G${}_{9}$, followed by giving $\left(c_{0}^{*},c_{1}^{*},c_{2}^{*}\prime \right)$ to $O_{sign}$. Then, $O_{sign}$ randomly chooses a message μ and $k\overset{\$}{\leftarrow }{\left\{0,1,2,3\right\}}^{n}$, and produces a signature (x,k) for μ. Next, $O_{sign}$ generates $c_{3}^{*}={\mathrm{Enc}}_{k}\left(\mu ,x\right)$ and gives it to C. Lastly, C gives $\left(c_{0}^{*},c_{1}^{*},c_{2}^{*},c_{3}^{*}\right)$ to A as the challenge ciphertext and waits to get (μ,k,x) from A.
- G${}_{11}$: C changes the challenge ciphertext a little. C does not give k to $O_{sign}$ and does not need to get $c_{3}$ from $O_{sign}$. C just gives the ciphertext $\left(c_{0}^{*},c_{1}^{*},c_{2}^{*}\right)$ generated by itself to A as the challenge ciphertext. If A is able to obtain the plaintext k in $c_{2}^{*}$ with non-negligible probability, C admits that A wins the game with the same probability.
- G${}_{12}$: This game is identical with the game G${}_{11}$ except that the challenge ciphertext $c_{1}^{*}$ is computed as $c_{1}^{*}=c_{0}^{*}p+e_{1}$ where $e_{1}\overset{\$}{\leftarrow }{\left(\left[-\varrho \sqrt{n},\varrho \sqrt{n}\right]⋂Z\right)}^{n}$.
- G${}_{13}$: C queries the variant RLWE oracle to fetch an instance $\left(\left(\begin{array}{c} a_{1}\\ a_{2} \end{array}\right),\left(\begin{array}{c} z_{1}\\ z_{2} \end{array}\right)\right)$ Then, C publishes the public keys as $h_{r}=a_{1},u=a_{2}$. C and sets the challenge ciphertext as follows $c_{0}^{*}=z_{1},c_{2}^{*}=z_{2}$. The construction method for $c_{1}^{*}$ remains identical with that in G${}_{12}$, that is $c_{1}^{*}=c_{0}^{*}p+e_{1}$.

□

**Lemma** **2.** *The games G${}_{1}$ and G${}_{0}$ are computationally indistinguishable when ϱ=20logn.*

**Proof.**  This lemma can be proven by hybrids. First, C chooses $h^{\prime }\overset{\$}{\leftarrow }R_{q}$ and calculates $b_{r}^{\prime }=h^{\prime }p+e$ where $p\overset{\$}{\leftarrow }{\left\{-1,0,1\right\}}^{n},e\overset{\$}{\leftarrow }{\left\{-\varrho ,\cdots ,\varrho \right\}}^{n}$. The infinity norm of the error e is set at 20logn, which satisfies the constraint for errors in RLWE, i.e., the Gaussian parameter $\alpha q\geq \omega (\sqrt{\log n})$ (see Proposition 1). According to the intractability of RLWE, $b_{r}^{\prime }$ and $b_{r}\overset{\$}{\leftarrow }R_{q}$ are computationally indistinguishable. Next, C continues modifying $b_{r}^{\prime }$ as $b_{r}^{\prime }=h_{r}p+e$. Since $h_{r}$ and $h^{\prime }$ are statistically indistinguishable according to the uniform property of the algorithm KeyGen, the new $b_{r}^{\prime }$ is computationally indistinguishable with $b_{r}\overset{\$}{\leftarrow }R_{q}$. Given all this, the games G${}_{1}$ and G${}_{0}$ are computationally indistinguishable.   □

**Lemma** **3.** *The games G${}_{2}$ and G${}_{1}$ are identical from the view of A.*

**Proof.**  Since the computation procedure for $\left(c_{0}^{*},c_{1}^{*},c_{2}^{*},c_{3}^{*}\right)$ is completely hidden from A. In the view of A, it makes no difference whether or not some challenge ciphertext has been generated before the public key is published. That is, the difference between G${}_{2}$ and G${}_{1}$ is only the difference in concept. Consequently, the lemma holds.    □

**Lemma** **4.** *The game G${}_{3}$ is statistically indistinguishable with the game G${}_{2}$.*

**Proof.**  The only difference between the games G${}_{3}$ and G${}_{2}$ is $b_{r}$. In G${}_{3}$, C calculates $b_{r}=h_{r}p+e-H_{0}\left(c_{0}^{*}\right)d_{r}$, while in G${}_{2}$$b_{r}=h_{r}p+e$. Since $c_{0}^{*}$ is fixed and $d_{r}$ submits to the uniform distribution. Therefore, the $b_{r}$ is statistically indistinguishable with the $b_{r}$ in the game G${}_{2}$.    □

Due to the uniform property of the public keys generated by the KeyGen algorithm, we have the following lemma.

**Lemma** **5.** *The game G${}_{4}$ is statistically indistinguishable with the game G${}_{3}$.*

It is difficult to directly prove the statistical indistinguishability between the games G${}_{5}$ and G${}_{4}$. However, the game sequences can be continued by the game transitions based on failure events.

**Lemma** **6.** *Let E${}_{5}$ denote the event that A makes the unsigncryption queries with the ciphertexts $\left(c_{0},c_{1},c_{2},c_{3}\right)$ meeting $c_{0}=c_{0}^{*}$ in phase I. From the point of view of A, the games G${}_{5}$ and G${}_{4}$ are totally the same, when E${}_{5}$ does not occur. Furthermore, $Pr\left[A_{5}|\neg E_{5}\right]=Pr\left[A_{4}|\neg E_{5}\right]$, and $Pr[E_{5}]$ is negligible.*

**Proof.**  First, in the game G${}_{5}$, C may reply to all the unsigncryption queries normally except for the queried ciphertexts $\left(c_{0},c_{1},c_{2},c_{3}\right)$ with $c_{0}=c_{0}^{*}$. Therefore, the behavior of C is identical in games G${}_{5}$ and G${}_{4}$, when E${}_{5}$ does not happen. That is, A learns exactly the same knowledge form C, when not considering the event E${}_{5}$.

Second, in the games G${}_{5}$ and G${}_{4}$, $c_{0}^{*}$ is hidden from A in phase I. Whether $c_{0}^{*}$ is obtained by evaluating $rh_{r}+e_{0}$ or by guessing, the probability that A computes a ciphertext with the form $\left(c_{0}^{*},c_{1},c_{2},c_{3}\right)$ is $q^{-n}$. That is to say, the probability that C can not correctly answer the valid unsigncryption queries is no more than $q^{-n}$, which is negligible. The difference is that C deliberately answers the query E${}_{5}$ with ⊥ in G${}_{5}$ instead of unsigncrypting normally as in G${}_{4}$. In summary, the reduction for the attack capability learned in G${}_{5}$ compared to that in G${}_{4}$ is negligible.    □

**Lemma** **7.** *Let E${}_{6}$ denote the events that A makes the unsigncryption queries with the ciphertexts $\left(c_{0},c_{1},c_{2},c_{3}\right)$ meeting $c_{0}\neq c_{0}^{*}$ but $H_{1}\left(c_{0}\right)=H_{1}\left(c_{0}^{*}\right)$ in phase I. The games G${}_{6}$ and G${}_{5}$ are identical in the adversary A’s view, when E${}_{6}$ does not happen, $Pr\left[A_{6}|\neg E_{6}\right]=Pr\left[A_{5}|\neg E_{6}\right]$. Moreover, $Pr[E_{6}]$ is negligible.*

**Proof.**  First, in G${}_{6}$, the approach of C to answer the unsigncryption queries is totally identical when the queried ciphertexts $\left(c_{0},c_{1},c_{2},c_{3}\right)$ satisfy $c_{0}\neq c_{0}^{*}$ and $H_{1}\left(c_{0}\right)=H_{1}\left(c_{0}^{*}\right)$. Therefore, the knowledge learned from G${}_{6}$ and G${}_{5}$ is the same, when not considering the event E${}_{6}$.

Second, for a known $c_{0}^{*}$, A finds a $c_{0}$ satisfying $c_{0}\neq c_{0}^{*}$ but $H_{0}\left(c_{0}\right)=H_{0}\left(c_{0}^{*}\right)$, which means A discovers a hash collision. We set the hash function to satisfy the security intensity of the proposed signcryption system, namely, the probability that hash collision occurs is no more than a negligible probability $2^{-\lambda }$. In fact, $c_{0}^{*}$ is hidden in phase I, then the probability that A just computes a ciphertext with $c_{0}\neq c_{0}^{*}$ and $H_{0}\left(c_{0}\right)=H_{0}\left(c_{0}^{*}\right)$ is also no more than the above probability $2^{-\lambda }$. Thus, even though the attack power of A obtained in G${}_{6}$ is less than that in G${}_{5}$, the difference is negligible.    □

**Lemma** **8.** *Let E${}_{7}$ denote the events that A set the unsigncryption queries with the type of ciphertext $\left(c_{0}^{*},c_{1}^{*},c_{2}^{*},c_{3}\right)$ meeting $c_{3}\neq c_{3}^{*}$ in phase II. In the adversary A’s view, the games G${}_{7}$ and G${}_{6}$ are identical, when E${}_{7}$ does not happen. Namely, $Pr\left[A_{7}|\neg E_{7}\right]=Pr\left[A_{6}|\neg E_{7}\right]$. Furthermore, $Pr[E_{7}]$ is negligible.*

**Proof.**  The ciphertext elements $\left(c_{0},c_{1},c_{2}\right)$ constitute the ciphertext of the public key encryption. Once the elements are determined, the plaintext k involved in the public key encryption is determined and unique. That is, the key used for the symmetric encryption is fixed. According to the one-to-one property of the symmetric encryption ENC, modifying $c_{3}$ will yield a distinct message–signature pair $\left(x^{\prime },\mu^{\prime }\right)={\mathrm{Dec}}_{k}\left(c_{3}\right)$ (relative to (x,μ)). The probability of the new message–signature pair successfully passing the signature verification is negligible. We prove this conclusion through a classification discussion. It can be divided into two cases.

- **Case 1**: A generates the ciphertext $c_{3}$ by its signature for some message μ. As an inner adversary, A has the ability to yield signatures by itself. However, the procedure to generate $c_{3}$ requires $k^{*}$. The probability of obtaining $k^{*}$ from $\left(c_{0}^{*},c_{1}^{*},c_{2}^{*}\right)$ is negligible due to the security of the public key encryption part. Please refer to Lemma 15 for more details.
- **Case 2**: A generates the ciphertext $c_{3}$ randomly. According to the one-to-one property of the symmetric encryption, a random message–signature pair (μ,x) is unsigncrypted from $c_{3}$. Since A does not possess knowledge of μ, it cannot generate a valid signature for it despite having the private key for signature generation. Consequently, the probability (μ,k,x) passing signature verification is negligible.

   □

**Lemma** **9.** *Let E${}_{8}$ denote the event that in phase II, A makes the unsigncryption queries with the form of the ciphertext as $\left(c_{0}^{*},c_{1},c_{2},c_{3}\right)$ and $\left(c_{1},c_{2}\right)\neq \left(c_{1}^{*},c_{2}^{*}\right)$. In the adversary A’s view, the games G${}_{8}$ and G${}_{7}$ are identical, when $E_{8}$ does not occur. Namely, $Pr\left[A_{8}|\neg E_{8}\right]=Pr\left[A_{7}|\neg E_{8}\right]$. Moreover, $Pr[E_{8}]$ is negligible.*

**Proof.**  This lemma can be argued by a classification discussion. Let $k,k^{*}$ be the keys concealed in $c_{2}$, $c_{2}^{*}$, respectively.

- **Case 1**: $k^{*}$ remains unchanged, i.e., $k=k^{*}$. To guarantee the validity of the ciphertext, the information hidden by $c_{2}$ should be $\left\lfloor \left(H\left(c_{1},c_{2}^{\prime }\right)⊕k^{*}\right)\left\lfloor q/8\right\rfloor /2^{\ell }\right\rfloor$. To fulfill this requirement, there are only two possible subcases.
  – A generates $c_{2}$ through encryption. On one hand, A does not know the $k^{*}$ concealed in $c_{2}^{*}$. On the other hand, A chooses a r distinct from the secret r used in the $c_{0}^{*}$, which will lead to an invalid public key ciphertext. Thus, the probability of this subcase occurring is negligible.
  – A produces $c_{2}$ by falsifying $c_{2}^{*}$. For this, A should have the ability to compute $c_{2}-c_{2}^{*}+\left\lfloor \left(\left(H\left(c_{1},c_{2}^{\prime }\right)⊕k^{*}\right)-\left(H\left(c_{1}^{*},c_{2}^{*}\prime \right)⊕k^{*}\right)\right)\left\lfloor q/8\right\rfloor /2^{\ell }\right\rfloor$. The probability of this event is negligible since $c_{2}^{*}\prime$ and k involved in $c_{2}^{*}$ are hidden from A.
- **Case 2**: $k\neq k^{*}$ and $c_{3}$ remains unchanged. In this case, due to the one-to-one property of the symmetric encryption, the message–signature pair (μ,k,x) extracted from $c_{3}$ is completely random. As a result, the probability of (μ,k,x) passing signature verification is negligible.
- **Case 3**: $k\neq k^{*}$ and $c_{3}\neq c_{3}^{*}$. The case can be divided into two subcases.
  – In the procedure to generate $c_{3}$, the plaintext μ corresponding to it is not known to A. Consequently, the probability $\left(c_{1}^{*},c_{2},c_{3}\right)$ being a valid ciphertext is negligible. The argument is the same as that of case 2 in the proof of Lemma 8.
  – The ciphertext $c_{3}$ is generated based on a valid message–signature pair produced by A. It can be further divided into two subcases. (1) $c_{2}$ is obtained though encrypting by A. The argument to this subcase is identical to subcase 1 of case 1 in this Lemma 9. Due to the uniqueness of LWE (see Lemma 1), r has already been determined by $c_{0}^{*}$. The probability of A choosing r used in $c_{0}^{*}$ is negligible. The the distinct r yields an invalid ciphertext. (2) $c_{2}$ is falsified from $c_{2}^{*}$ by A. This is similar to the demonstration in subcase 2 of case 1 of Lemma 9. A needs to compute $c_{2}-c_{2}^{*}+\left\lfloor \left(\left(H\left(c_{1},c_{2}^{\prime }\right)⊕k\right)-\left(H\left(c_{1}^{*},c_{2}^{*}\prime \right)⊕k^{*}\right)\right)\left\lfloor q/8\right\rfloor /2^{\ell }\right\rfloor$ without knowing $k^{*}$, and the probability of this event is negligible.

   □

**Algorithm 1** SimExtractE$\left(h_{r},b_{r},d_{r},\tau ,\tau^{*},u\right)$**Require:** public keys $h_{r},b_{r},d_{r}\in R_{q}$, in which $d_{r}\leftarrow \mathrm{KeyGen}$, $b_{r}=h_{r}p+e$ for $p,e\leftarrow {\left\{-\varrho ,\cdots ,\varrho \right\}}^{n}$; a pair of un-equivalent tags $\tau ,\tau^{*}\leftarrow {\left\{-\varrho ,\cdots ,\varrho \right\}}^{n}$; a syndrome $u\in R_{q}$.**Ensure:**  A private key $\left(x_{1},x_{2}\right)$.
 1:$x_{0}^{\prime }\leftarrow {\left\{-1,0,1\right\}}^{n}$ 2:$x^{\prime }=\left(u-h_{r}x_{0}^{\prime }\right)/\left(\tau -\tau^{*}\right)$ 3:$\left(x_{1}^{\prime },x_{1}\right)=\left(x^{\prime },0\right)-\mathrm{SamplePre}\left(B_{d},\eta_{2},\left(x^{\prime },0\right)\right)$ ($\eta_{2}=\frac{1}{\sqrt{2}\pi^{2}}n{\left(\ln\left(2^{(7+\lambda )/2}n\right)\right)}^{3/2}$) 4:$x_{0}=x_{0}^{\prime }-px_{1}$ 5:output $\left(x_{0},x_{1}\right)$ as the private key.

**Lemma** **10.** *Under the parameter settings as in Equation (1), the $\left(x_{0},x_{1}\right)$ generated in Algorithm 1 is a valid private key. That is, $∥u-\left[h_{r}∥b_{r}+\tau d_{r}\right]\left(x_{0},x_{1}\right)∥\;\leq \;\frac{3}{\sqrt{2}\pi^{2}}\varrho n^{5/2}{\left(\ln\left(2^{(7+\lambda )/2}n\right)\right)}^{3/2}$ and $∥\left(x_{0},x_{1}\right)∥\;\leq \;\frac{1}{\sqrt{2}\pi^{2}}\varrho n^{5/2}{\left(\ln\left(2^{(7+\lambda )/2}n\right)\right)}^{3/2}$. It ensures that this private key can be used correctly in unsigncryption.*

**Proof.**  First, we argue that the multiplication of $\left[h_{r}∥b_{r}+\tau d_{r}\right]$ and $\left(x_{0},x_{1}\right)$ is close enough to u.

(2) $$\begin{array}{c} \begin{array}{cc} \left[h_{r}∥b_{r}+\tau d_{r}\right]\left(x_{0},x_{1}\right) & =h_{r}x_{0}+\left(h_{r}p+e-\tau^{*}d_{r}+\tau d_{r}\right)x_{1}\\ \; & =h_{r}\left(x_{0}+px_{1}\right)+ex_{1}+\left(\tau -\tau^{*}\right)d_{r}x_{1}\\ \; & =h_{r}x_{0}^{\prime }+ex_{1}+\left(\tau -\tau^{*}\right)\left(x^{\prime }-x_{1}^{\prime }\right)\\ \; & =h_{r}x_{0}^{\prime }+ex_{1}+\left(u-h_{r}x_{0}^{\prime }\right)-\left(\tau -\tau^{*}\right)x_{1}^{\prime }\\ \; & =u+ex_{1}-\left(\tau -\tau^{*}\right)x_{1}^{\prime } \end{array} \end{array}$$

(3) $$\begin{array}{c} \begin{array}{cc} ∥u-\left[h_{r}∥b_{r}+\tau d_{r}\right]\left(x_{0},x_{1}\right)∥= & ∥\left(\tau -\tau^{*}\right)x_{1}^{\prime }-ex_{1}∥\;\leq \;∥ex_{1}∥\;+\;∥\left(\tau -\tau^{*}\right)x_{1}^{\prime }∥\\ \leq & {∥e∥}_{\infty }∥x_{1}∥\sqrt{n}\sqrt{n}+∥Ø-{Ø}^{*}{∥}_{\infty }∥x_{1}^{\prime }∥\sqrt{n}\sqrt{n}\\ = & \varrho \eta_{2}\sqrt{n}n+2\eta_{2}\sqrt{n}n\leq \left(\varrho +2\right)\frac{1}{\sqrt{2}\pi^{2}}n{\left(\ln\left(2^{(7+\lambda )/2}n\right)\right)}^{3/2}n^{3/2}\\ = & \frac{1}{\sqrt{2}\pi^{2}}\left(\varrho +2\right)n^{5/2}{\left(\ln\left(2^{(7+\lambda )/2}n\right)\right)}^{3/2}. \end{array} \end{array}$$

Second, we demonstrate that $\left(x_{0},x_{1}\right)$ is short.

(4) $$\begin{array}{c} \begin{array}{cc} ∥(x_{0},x_{1})∥= & \sqrt{∥x_{0}{∥}^{2}+{∥x_{1}∥}^{2}}\leq \sqrt{∥x_{0}^{\prime }{∥}^{2}+∥px_{1}{∥}^{2}+{∥x_{1}∥}^{2}}\\ \approx & ∥px_{1}{∥\;\leq \;∥p∥}_{\infty }∥x_{1}∥\sqrt{n}\sqrt{n}\approx \varrho \eta_{2}\sqrt{n}n\\ \leq & \frac{1}{\sqrt{2}\pi^{2}}n\varrho {\left(\ln\left(2^{(7+\lambda )/2}n\right)\right)}^{3/2}n^{3/2}\\ = & \frac{1}{\sqrt{2}\pi^{2}}\varrho n^{5/2}{\left(\ln\left(2^{(7+\lambda )/2}n\right)\right)}^{3/2} \end{array} \end{array}$$

Here, $x_{0}$ takes up most of the length of the whole vector $\left[x_{0},x_{1}\right]$. That is,

(5) $$\begin{array}{c} \begin{array}{c} ∥x_{0}∥\;\leq \;\frac{1}{\sqrt{2}\pi^{2}}\varrho n^{5/2}{\left(\ln\left(2^{(7+\lambda )/2}n\right)\right)}^{3/2},\;\;∥x_{1}∥\leq \frac{1}{\sqrt{2}\pi^{2}}n^{3/2}{\left(\ln\left(2^{(7+\lambda )/2}n\right)\right)}^{3/2}. \end{array} \end{array}$$

From the above, the private key and the error are of the same magnitude, differing only by a constant factor. In fact, we can use Lemma 3 of [43] to reduce the length of the private key and the error by the factor $\sqrt{n}$, so that the modulus *q* can also be reduced by $\sqrt{n}$.    □

**Lemma** **11.** *In the adversary A’s view, the games G${}_{9}$ and G${}_{8}$ are totally identical. Furthermore, $Pr\left[F_{9}\right]=Pr\left[F_{8}\right]$.*

**Proof.**  First, the public keys in the games G${}_{9}$ and G${}_{8}$ are identical. Second, C does not reply to the unsigncryption queries for the ciphertexts with $c_{0}\neq c_{0}^{*}$ in the games G${}_{9}$ and G${}_{8}$. Third, we argue that C can correctly unsigncrypt with the private key produced in Algorithm 1, when $c_{0}\neq c_{0}^{*}$. Let $\tilde{c_{2}}=ru+e_{2}+\left(H_{2}\left(c_{1},c_{2}^{\prime }\right)⊕k\right)\left\lfloor q/8\right\rfloor$.

(6) $$\begin{array}{c} \begin{array}{cc} 2^{\ell }c_{2}-\left[c_{0}∥c_{1}\right]\left(x_{0},x_{1}\right) & =2^{\ell }c_{2}-\tilde{c_{2}}+\tilde{c_{2}}-\left[c_{0}∥c_{1}\right]\left(x_{0},x_{1}\right)\\ \; & =1-2^{\ell }+ru+e_{2}+k\left\lfloor q/8\right\rfloor -\left[\left(rh_{r}+e_{0}\right)∥\left(r\left(b_{r}+H_{1}\left(c_{0}\right)d_{r}\right)+e_{1}\right)\right]\left(x_{0},x_{1}\right)\\ \; & \approx k\left\lfloor q/8\right\rfloor +r\left[u-\left[h_{r}∥\left(b_{r}+H_{1}\left(c_{0}\right)d_{r}\right)\right]\left(x_{0},x_{1}\right)\right]+e_{2}-e_{0}x_{0}-e_{1}x_{1} \end{array} \end{array}$$

Let $e^{\prime }=u-\left[h_{r}∥\left(b_{r}+H_{1}\left(c_{0}\right)d_{r}\right)\right]\left(x_{0},x_{1}\right)$. According to Lemma 10, $∥e^{\prime }∥\;\leq \;\frac{1}{\sqrt{2}\pi^{2}}\left(\varrho^{2}+3\varrho \right)n^{5/2}{\left(\ln\left(2^{(7+\lambda )/2}n\right)\right)}^{3/2}$. Then,

(7) $$\begin{array}{c} \begin{array}{cc} \; & ∥r\left[u-\left[h_{r}∥\left(b_{r}+H_{1}\left(c_{0}\right)d_{r}\right)\right]\left(x_{0},x_{1}\right)\right]+e_{2}-e_{0}x_{0}-e_{1}x_{1}{∥}_{\infty }={∥re^{\prime }+e_{2}-e_{0}x_{0}-e_{1}x_{1}∥}_{\infty }\\ \leq & ∥re^{\prime }{∥}_{\infty }+∥e_{2}{∥}_{\infty }+∥e_{0}x_{0}{∥}_{\infty }+∥e_{1}x_{1}{∥}_{\infty }\leq {∥r∥}_{\infty }∥e^{\prime }∥\sqrt{n}+∥e_{2}{∥}_{\infty }+∥e_{0}{∥}_{\infty }∥x_{0}∥\sqrt{n}+∥e_{1}{∥}_{\infty }∥x_{1}∥\sqrt{n}\\ \leq & ∥e^{\prime }∥\sqrt{n}+∥e_{2}{∥}_{\infty }+∥e_{0}{∥}_{\infty }∥x_{0}^{\prime }∥\sqrt{n}+∥e_{0}{∥}_{\infty }{∥p∥}_{\infty }∥x_{1}∥\sqrt{n}\sqrt{n}\sqrt{n}+∥e_{1}{∥}_{\infty }∥x_{1}∥\sqrt{n}\\ \leq & \frac{3}{\sqrt{2}\pi^{2}}\varrho n^{5/2}{\left(\ln\left(2^{(7+\lambda )/2}n\right)\right)}^{3/2}\sqrt{n}+\varrho +\varrho \sqrt{n}\sqrt{n}+\varrho^{2}\frac{1}{\sqrt{2}\pi^{2}}n{\left(\ln\left(2^{(7+\lambda )/2}n\right)\right)}^{3/2}n^{1/2}n^{3/2}\\ \; & +\varrho \sqrt{n}\frac{1}{\sqrt{2}\pi^{2}}n\ln{\left(2^{(7+\lambda )/2}n\right)}^{3/2}n^{1/2}n^{1/2}\\ \approx & \frac{1}{\sqrt{2}\pi^{2}}\left(\varrho^{2}+3\varrho \right)n^{3}{\left(\ln\left(2^{(7+\lambda )/2}n\right)\right)}^{3/2} \end{array} \end{array}$$

Since $\frac{1}{\sqrt{2}\pi^{2}}\left(\varrho^{2}+3\varrho \right)n^{3}{\left(\ln\left(2^{(7+\lambda )/2}n\right)\right)}^{3/2}<\frac{1}{9}q$, the simulated private key can be used to correctly decrypt the public-key ciphertext. In summary, the games G${}_{9}$ and G${}_{8}$ are identical in A’s view. Therefore, A has the same ability to attack the proposed schemes in G${}_{9}$ and G${}_{8}$.    □

**Lemma** **12.** *In the adversary A’s view, the games G${}_{10}$ and G${}_{9}$ are identical. It is reasonable that C needs A to return (μ,k,x). Moreover, $Pr\left[F_{10}\right]=Pr\left[F_{9}\right]$.*

**Proof.**  In the games G${}_{10}$ and G${}_{9}$, the difference is that μ is chosen by $O_{sign}$, and the signature (k,x) and the ciphertext $c_{3}^{*}$ are also generated by $O_{sign}$ in G${}_{10}$, while they are all generated by C in G${}_{9}$. However, the public keys and private keys for the signature are identical, and the procedure for generating (μ,k,x) is executed strictly according to the signcryption algorithm. Therefore, the distribution of the challenge ciphertexts and the signatures involved in them are also identical in both games. In a word, the difference between the two games is only conceptual, and A’s view in the games G${}_{10}$ and G${}_{9}$ are completely the same. Therefore, what A can learn in the two games is identical.

Next, we demonstrate by contradiction that it is reasonable to require A to return (μ,k,x) in G${}_{10}$. Suppose that A does not know the information of k, but it can recover (μ,x). That is, A computes the correct μ without knowing $\left(c_{0}^{*},c_{1}^{*},c_{2}^{*}\right)$. Due to $\left(\mu ,x\right)=DEC_{K}\left(c_{3}\right)$, A obtaining (μ,x) from $c_{3}$ without k is contradictory to the security of the symmetric encryption. That is, A knows k with the same probability to recover (μ,x). Therefore, it is equivalent to computing (μ,x) and computing (μ,k,x) from the challenge ciphertext. Thus, C requiring A to return (μ,k,x) does not increase the hardness to attack the scheme.    □

**Lemma** **13.** *The modification to the game is rational, and the hardness of winning the game G${}_{11}$ is the same as that of winning G${}_{10}$, in A’s view. Furthermore, $Pr\left[F_{11}\right]=Pr\left[F_{10}\right]$.*

**Proof.**  First, as proven in Lemma 12, if A can guess the k encrypted in $c_{2}$, then it can compute the corresponding message and signature when given $c_{3}$. From this perspective, the crux of cracking the proposed scheme lies in obtaining k.

Second, $c_{3}$ does not provide any assistance in obtaining k. Firstly, it is impossible to directly obtain k from $c_{3}$. Since the symmetric encryption used in this scheme is IND-OT secure and one-to-one, $c_{3}$ does not leak any information of $H_{3}\left(k\right)$ to A. Therefore, $c_{3}$ does not disclose any information of k. Secondly, as proven in Lemma 8, the probability of obtaining assistance in computing k by changing $c_{3}$ is negligible.

Third, in the absence of $c_{3}$, it will not increase the probability of obtaining k by falsifying $\left(c_{0}^{*},c_{1}^{*},c_{2}^{*}\right)$. Although the message and signature (μ,x) concealed in $c_{3}$ can help to verify the correctness of k, the information of k can not also be changed without $c_{3}$. As proven in Lemma 7, the probability that A gets assistance to obtain k by changing $c_{0}^{*}$ is negligible. The probability of A receiving assistance to obtain k by changing $\left(c_{1}^{*},c_{2}^{*}\right)$ is also negligible, as proven in Lemma 9.

In conclusion, the hardness of winning the games G${}_{11}$ and G${}_{10}$ is identical whether $c_{3}$ is known or not.    □

**Lemma** **14.** *In A’s view, the games G${}_{12}$ and G${}_{11}$ are statistically indistinguishable. Furthermore, $Pr\left[F_{12}|\neg E_{12}\right]=Pr\left[F_{11}|\neg E_{11}\right]$ and $Pr\left[E_{12}\right]=Pr\left[E_{11}\right]$ is negligible.*

**Proof.**  In the game G${}_{11}$, $c_{1}^{*}=r\left(b_{r}+H_{1}\left(c_{0}^{*}\right)d_{r}\right)+e_{1}=r\left(h_{r}p+e-H_{1}\left(c_{0}^{*}\right)d_{r}+H_{1}\left(c_{0}^{*}\right)d_{r}\right)+e_{1}=rh_{r}p+re+e_{1}=c_{0}^{*}p-e_{0}p+re+e_{1}$.

$$\begin{array}{c} \begin{array}{cc} c_{1}^{*} & =r\left(b_{r}+H_{1}\left(c_{0}^{*}\right)d_{r}\right)+e_{1}=r\left(h_{r}p+e-H_{1}\left(c_{0}^{*}\right)d_{r}+H_{1}\left(c_{0}^{*}\right)d_{r}\right)+e_{1}\\ \; & =rh_{r}p+re+e_{1}=c_{0}^{*}p-e_{0}p+re+e_{1} \end{array} \end{array}$$

Let $\tilde{c_{1}^{*}}$ denote the ciphertext $c_{1}^{*}$ in the game G${}_{12}$. Then, $\tilde{c_{1}^{*}}=c_{0}^{*}p+e_{1}=c_{1}^{*}+e_{0}p-re$, where $c_{1}^{*}$ is the corresponding ciphertext in G${}_{11}$. We need to argue that $\left(c_{0}^{*},\tilde{c_{1}^{*}},c_{2}^{*}\right)$ is also a valid ciphertext. That is, $\left(c_{0}^{*},\tilde{c_{1}^{*}},c_{2}^{*}\right)$ can be decrypted correctly with the valid private key. Suppose that $\left(s_{0},s_{1}\right)$ is a valid private key for the ciphertext, i.e., $\left(s_{0},s_{1}\right)$ is short enough. It might be good to set the key $\left(s_{0},s_{1}\right)$ to an equal element size to that of the simulated key. The simulated key can decrypt well, although in the two kinds of known keys, the element size of the simulated key is a little larger than that of the real key. That is, $∥s_{0}∥\;\leq \;\frac{1}{\sqrt{2}\pi^{2}}\varrho n^{5/2}{\left(\ln\left(2^{(7+\lambda )/2}n\right)\right)}^{3/2}$, $∥s_{1}∥\;\leq \;\frac{1}{\sqrt{2}\pi^{2}}\varrho n^{3/2}{\left(\ln\left(2^{(7+\lambda )/2}n\right)\right)}^{3/2}$.

(8) $$\begin{array}{c} \begin{array}{cc} ∥c_{2}^{*}-\left(c_{0}^{*}∥\tilde{c_{1}^{*}}\right)\left(s_{0}∥s_{1}\right){∥}_{\infty }= & ∥c_{2}^{*}-\left(c_{0}^{*}∥c_{1}^{*}\right)\left(s_{0}∥s_{1}\right)-\left(\tilde{c_{1}^{*}}-c_{1}^{*}\right)s_{1}{∥}_{\infty }\\ \leq & ∥c_{2}^{*}-\left(c_{0}^{*}∥c_{1}^{*}\right)\left(s_{0}∥s_{1}\right){∥}_{\infty }+{∥\left(\tilde{c_{1}^{*}}-c_{1}^{*}\right)s_{1}∥}_{\infty }. \end{array} \end{array}$$

(9) $$\begin{array}{c} \begin{array}{cc} ∥c_{2}^{*}-\left(c_{0}^{*}∥\tilde{c_{1}^{*}}\right)\left(s_{0}∥s_{1}\right){∥}_{\infty }\; & =∥c_{2}^{*}-\left(c_{0}^{*}∥c_{1}^{*}\right)\left(s_{0}∥s_{1}\right)-\left(\tilde{c_{1}^{*}}-c_{1}^{*}\right)s_{1}{∥}_{\infty }\\ \; & \leq ∥c_{2}^{*}-\left(c_{0}^{*}∥c_{1}^{*}\right)\left(s_{0}∥s_{1}\right){∥}_{\infty }+{∥\left(\tilde{c_{1}^{*}}-c_{1}^{*}\right)s_{1}∥}_{\infty } \end{array} \end{array}$$

$∥c_{2}^{*}-\left(c_{0}^{*}∥c_{1}^{*}\right)\left(s_{0}∥s_{1}\right){∥}_{\infty }\leq \frac{1}{\sqrt{2}\pi^{2}}\left(\varrho^{2}+3\varrho \right)n^{3}{\left(\ln\left(2^{(7+\lambda )/2}n\right)\right)}^{3/2}$.

(10) $$\begin{array}{c} \begin{array}{cc} ∥\left(\tilde{c_{1}^{*}}-c_{1}^{*}\right)s_{1}{∥}_{\infty } & =∥\left(e_{0}p-re\right)s_{1}{∥}_{\infty }\leq ∥e_{0}ps_{1}{∥}_{\infty }+∥res_{1}{∥}_{\infty }\leq ∥e_{0}{∥}_{\infty }∥ps_{1}∥\sqrt{n}+{∥r∥}_{\infty }{∥es_{1}∥}_{\infty }\\ \; & \leq {\varrho ∥p∥}_{\infty }∥s_{1}∥n^{3/2}+{∥e∥}_{\infty }∥s_{1}∥n^{3/2}\leq \left(\varrho^{2}+\varrho \right)\frac{1}{\sqrt{2}\pi^{2}}n{\left(\ln\left(2^{(7+\lambda )/2}n\right)\right)}^{3/2}\sqrt{n}n^{3/2}\\ \; & \leq \frac{1}{\sqrt{2}\pi^{2}}\left(\varrho^{2}+\varrho \right){\left(\ln\left(2^{(7+\lambda )/2}n\right)\right)}^{3/2}n^{3} \end{array} \end{array}$$

Therefore, $∥c_{2}^{*}-\left(c_{0}^{*}∥\tilde{c_{1}^{*}}\right)\left(s_{0}∥s_{1}\right){∥}_{\infty }\leq \frac{1}{\sqrt{2}\pi^{2}}\left(2\varrho^{2}+4\varrho \right){\left(\ln\left(2^{(7+\lambda )/2}n\right)\right)}^{3/2}n^{3}<\left\lfloor q/16\right\rfloor$. Thus, the challenge ciphertext is also a valid one. That is, if A can obtain the correct k from $\left(c_{0}^{*},c_{1}^{*},c_{2}^{*}\right)$, then it can also obtain the same k from $\left(c_{0}^{*},\tilde{c_{1}^{*}},c_{2}^{*}\right)$.    □

**Lemma** **15.** *In A’s view, the games G${}_{13}$ and G${}_{12}$ are computationally indistinguishable. Furthermore, $Pr\left[F_{13}|\neg E_{13}\right]=Pr\left[F_{12}|\neg E_{12}\right]$, and $Pr\left[E_{13}\right]=Pr\left[E_{12}\right]$ is negligible.*

**Proof.**  Reduction from LWE. When the LWE oracle is $O=O_{s}$, namely the pseudorandom case, the challenge ciphertext has the same distribution as in the game G${}_{12}$. First, the public keys used directly to encrypt the challenge ciphertext are $\left(h_{r},b_{r}+H_{1}\left(c_{0}^{*}\right)d_{r},u\right)=\left(a_{1},a_{1}p+e-H_{1}\left(c_{0}^{*}\right)d_{r}+H_{1}\left(c_{0}^{*}\right)d_{r},a_{2}\right)=\left(a_{1},a_{1}p+e,a_{2}\right)$. Second, the challenge ciphertext is $c_{0}^{*}=z_{0}=ra_{1}+e_{0}$ for some $r,e_{0}\leftarrow {\left\{-1,0,1\right\}}^{n}$, according to the definition of LWE. Due to $z_{2}=ra_{2}+e_{2}$, $c_{2}^{*}=z_{2}+k\left\lfloor q/2\right\rfloor =ra_{2}+e_{2}+k\left\lfloor q/2\right\rfloor$. The $c_{1}^{*}$ is as follows.

$$c_{1}^{*}=c_{0}^{*}p+\tilde{e}=\left(ra_{1}+e_{0}\right)p+\left(re-e_{0}p+e_{1}\right)=r\left(a_{1}r+e\right)+e_{1}=r\left(b_{r}+H_{1}\left(c_{0}^{*}\right)d_{r}\right)+e_{1}.$$

Obviously, the challenge ciphertext $c^{*}$ is exactly the challenge ciphertext in game G${}_{12}$. In this case, A’s advantage to distinguish the two games is zero.

When the LWE oracle is the real random case $O=O_{\$}$, the challenge ciphertext is uniformly distributed. In the challenge ciphertext, $c_{0}^{*}=z_{0}$ obeys the uniform distribution. Due to $z_{1}\overset{\$}{\leftarrow }Z_{q}^{n}$, $c_{2}^{*}=z_{1}+k\left\lfloor q/2\right\rfloor$ is distributed uniformly. On the other hand, the generation method for $c_{1}^{*}$ stays the same as in game G${}_{12}$. $c_{1}^{*}=\left(z_{1}p+e_{1}\right)-e_{0}p+re$. $\left(z_{1}p+e_{1}\right)$ is computationally indistinguishable from the uniform distribution over $Z_{q}^{n}$, and $-e_{0}p+re$ is restricted to a small range. Therefore, $c_{1}^{*}$ is computationally indistinguishable from the uniform distribution over $Z_{q}^{n}$. That is to say, in terms of distinguishing the games G${}_{12}$ and G${}_{11}$, $c_{1}^{*}$ is no more effective than $\left(c_{0}^{*},c_{2}^{*}\right)$.

C uses the guess from A as the answer for the LWE oracle. Therefore, the advantage of A in distinguishing the two games is an adversary’s advantage in attacking RLWE, which is negligible.    □

In Theorem 2, the unforgeability of SC-NTRU will be proven by the interaction between the challenger C and an adversary A. To complete Theorem 2, some necessary conclusions are demonstrated in Lemmas 16 to 18.

**Lemma** **16.** *In the query phase, the simulated signature generated by the simulated trapdoor in Algorithm 2 is indistinguishable from the real signature, and it can pass the signature verification.*

**Proof.**  To argue that the signature generated by the simulated trapdoor in Algorithm 2 and that by the real trapdoor are identical, it is sufficient to demonstrate that the simulated trapdoor is short and has the same structure property as that of the real trapdoor. For the size property, we argue that the simulated trapdoor is short enough that it can use the same Gaussian parameter to sample as that used in the sample with the real trapdoor. For the structure property, we show that the size of the deviation and the length of the simulated trapdoor are the same, which is also the inherent character of the real trapdoor.

In Algorithm 2, for the case of r=0:

(11) $$\begin{array}{c} \begin{array}{cc} \left[h_{s}∥f\right]\left(x_{0},x_{1}\right) & =\left[h_{s}∥h_{s}p+e+ph_{f}\right]\left(x_{0}^{\prime }-px_{1},x_{1}\right)=h_{s}\left(x_{0}^{\prime }-px_{1}\right)+\left(h_{s}p+e+ph_{f}\right)x_{1}\\ \; & =p\left(h_{s}x_{0}^{\prime }/p+h_{f}x_{1}\right)+ex_{1}=ex_{1}-px_{1}^{\prime }\\ ∥ex_{1}-px_{1}^{\prime }∥ & \leq p∥x_{1}^{\prime }∥+∥ex_{1}∥\leq p∥x_{1}^{\prime }{∥+∥e∥}_{\infty }∥x_{1}∥\sqrt{n}\sqrt{n}\leq p\eta_{3}^{\prime }\sqrt{n}+\varrho \left(ℵ+1\right)\eta_{3}^{\prime }\sqrt{n}\sqrt{n}\sqrt{n}\\ \; & =\eta_{3}^{\prime }\sqrt{n}\left(p+\varrho \left(ℵ+1\right)n\right)=\eta_{3}^{\prime }\sqrt{n}\left(ℵ+1\right)\left(\xi +\varrho n\right)\approx \varrho \left(ℵ+1\right)\frac{1}{\sqrt{2}\pi^{2}}\sqrt{n}\ln\left(2^{(7+\lambda )/2}n\right)n^{3/2}\\ \; & =\frac{1}{\sqrt{2}\pi^{2}}\varrho \left(ℵ+1\right)n^{2}\ln\left(2^{(7+\lambda )/2}n\right)\\ ∥(x_{0},x_{1})∥ & =∥\left(x_{0}^{\prime }-px_{1},x_{1}\right)∥=\sqrt{∥x_{0}^{\prime }-px_{1}{∥}^{2}+{∥x_{1}∥}^{2}}\leq \sqrt{∥x_{0}^{\prime }{∥}^{2}+∥px_{1}{∥}^{2}+{∥x_{1}∥}^{2}}\\ \; & \approx ∥px_{1}{∥\leq ∥p∥}_{\infty }∥x_{1}∥\sqrt{n}\sqrt{n}\leq \left(ℵ+1\right)\eta_{3}n^{3/2}=\frac{1}{\sqrt{2}\pi^{2}}\left(ℵ+1\right)n^{2}\ln\left(2^{(7+\lambda )/2}n\right) \end{array} \end{array}$$

In the case of r=1:

(12) $$\begin{array}{c} \begin{array}{cc} \left[h_{s}∥f\right]\left(x_{0},x_{1}\right) & =\left[h_{s}∥h_{s}p+e+ph_{f}\right]\left(-px_{1},x_{1}\right)=h_{s}\left(-px_{1}\right)+\left(h_{s}p+e+ph_{f}\right)x_{1}\\ \; & =p\left(x_{1}^{\prime }+h_{f}x_{1}\right)+ex_{1}-px_{1}^{\prime }=ex_{1}-px_{1}^{\prime }\\ ∥ex_{1}-px_{1}^{\prime }∥ & \leq p∥x_{1}^{\prime }∥+∥ex_{1}∥\leq p∥x_{1}^{\prime }{∥+∥e∥}_{\infty }∥x_{1}∥\sqrt{n}\sqrt{n}\leq p\eta_{0}\sqrt{n}+\varrho \left(ℵ+1\right)\eta_{0}\sqrt{n}n\\ \; & =\eta_{0}\sqrt{n}\left(\left(ℵ+1\right)\xi +\varrho \left(ℵ+1\right)n\right)\approx \frac{1}{\sqrt{2}\pi }\varrho \left(ℵ+1\right)n^{3/2}\sqrt{\ln\left(2^{(7+\lambda )/2}n\right)}\\ ∥(x_{0},x_{1})∥ & =∥\left(-px_{1},x_{1}\right)∥\leq \sqrt{∥px_{1}{∥}^{2}+{∥x_{1}∥}^{2}}\approx {∥p∥}_{\infty }∥x_{1}∥\sqrt{n}\sqrt{n}=\left(ℵ+1\right)\eta_{0}\sqrt{n}n\\ \; & \approx \frac{1}{\sqrt{2}\pi }\left(ℵ+1\right)n^{3/2}\sqrt{\ln\left(2^{(7+\lambda )/2}n\right)} \end{array} \end{array}$$

When using the simulated trapdoor to sign, the Gaussian parameter to sample is

$$\frac{1}{\sqrt{2}\pi^{2}}\left(ℵ+1\right)n^{2}\ln\left(2^{(7+\lambda )/2}n\right)\frac{1}{\sqrt{2}\pi }\sqrt{\ln\left(2^{(7+\lambda )/2}n\right)}=\frac{1}{2\pi^{3}}\left(ℵ+1\right)n^{2}{\left(\ln\left(2^{(7+\lambda )/2}n\right)\right)}^{3/2}$$

For simplicity, it can be set as $\frac{1}{2\pi^{3}}\left(ℵ+1\right)n^{2}{\left(\ln\left(2^{(7+\lambda )/2}n\right)\right)}^{3/2}$. The Gaussian parameter for the real trapdoor to sign is also this value, according to Equation (1). It is easy to see that the size and structure property of the simulated trapdoor are the same as those of the real trapdoor, respectively.    □

**Lemma** **17.** *Under the parameter settings in Equation (1), the vector $x_{0}+px_{1}$ constructed by C in the forgery phase of Theorem 2 derives a valid solution for the search RLWE instance $\left(h_{s},y\right)$.*

**Proof.**  Since the x output by A is a valid forgery, x obeys $D_{Z^{n},0,\eta }$ with $\eta =\frac{1}{2\pi^{3}}\left(ℵ+1\right)n^{2}{\left(\ln\left(2^{(7+\lambda )/2}n\right)\right)}^{3/2}$ and $∥y-\left(h_{s},f\right){x∥}_{\infty }\leq \frac{1}{2\pi^{3}}\left(ℵ+1\right)\varsigma n^{2}{\left(\ln\left(2^{(7+\lambda )/2}n\right)\right)}^{3/2}$ for some constant ς>1. Then, it has

(13) $$\begin{array}{c} \begin{array}{cc} \; & ∥y-h_{s}\left(x_{0}+px_{1}\right){∥}_{\infty }=∥y-[h_{s}\left|\right|h_{s}p]\left(x_{0},x_{1}\right){∥}_{\infty }=∥y-[h_{s}\left|\right|h_{s}p+e{]\left(x_{0},x_{1}\right)+ex_{1}∥}_{\infty }\\ \leq & ∥y-[h_{s}\left|\right|h_{s}p+e]\left(x_{0},x_{1}\right){∥}_{\infty }+∥ex_{1}{∥}_{\infty }\leq \frac{1}{2\pi^{3}}\left(ℵ+1\right)\varsigma n^{2}{\left(\ln\left(2^{(7+\lambda )/2}n\right)\right)}^{3/2}+{∥e∥}_{\infty }∥x_{1}∥\sqrt{n}\\ \leq & \frac{1}{2\pi^{3}}\left(ℵ+1\right)\varsigma n^{2}{\left(\ln\left(2^{(7+\lambda )/2}n\right)\right)}^{3/2}+\varrho \frac{1}{2\pi^{3}}{\left(ℵ+1\right)}^{2}n^{2}{\left(\ln\left(2^{(7+\lambda )/2}n\right)\right)}^{3/2}n\\ \approx & \frac{1}{2\pi^{3}}\varrho {\left(ℵ+1\right)}^{2}n^{3}{\left(\ln\left(2^{(7+\lambda )/2}n\right)\right)}^{3/2} \end{array} \end{array}$$

Similarly, $∥y-h_{s}\left(x_{0}+px_{1}\right)∥\leq \frac{1}{2\pi^{3}}\varrho {\left(ℵ+1\right)}^{2}n^{7/2}{\left(\ln\left(2^{(7+\lambda )/2}n\right)\right)}^{3/2}$.

Furthermore, the size of the vector $x_{0}+px_{1}$ may be evaluated in the same approach.

(14) $$\begin{array}{c} \begin{array}{cc} ∥x_{0}+px_{1}∥\leq & ∥x_{0}∥+∥px_{1}∥\leq ∥x_{0}{∥+∥p∥}_{\infty }∥x_{1}∥\sqrt{n}\sqrt{n}\\ \leq & \frac{1}{2\pi^{3}}{\left(ℵ+1\right)}^{2}n^{7/2}{\left(\ln\left(2^{(7+\lambda )/2}n\right)\right)}^{3/2}. \end{array} \end{array}$$

Therefore, $y-h_{s}\left(x_{0}+px_{1}\right)$ and $x_{0}+px_{1}$ is short enough, and they form a solution for the RLWE instance $\left(h_{s},y\right)$ under the parameter settings in Equation (1).    □

**Lemma** **18.** *For the query times $0<Q<\sqrt{\frac{2}{3}\pi ℵ\xi \left(\xi +1\right)}$, the upper bound ξ of $p_{i}$ for i from 1 to ℵ. Both the signature queries and forgery in the simulation can be completed without aborts, with probability*

(15) $$\begin{array}{c} \begin{array}{c} \frac{\sqrt{3}}{\pi \sqrt{2ℵ\xi (\xi +1)}}\left(1-\frac{\sqrt{3}Q}{\pi \sqrt{2ℵ\xi (\xi +1)}}\right)\leq Pr\left[complete\right]\leq \frac{\sqrt{3}}{\pi \sqrt{2ℵ\xi (\xi +1)}}, \end{array} \end{array}$$

*no matter what strategy A takes. In particular, when $Q<\frac{\sqrt{3}\pi^{4}q}{20n^{2}{ℵ}^{1/2}{\left(\ln\left(2^{(7+\lambda )/2}n\right)\right)}^{3/2}}$, the sucessful probability satisfies $\frac{\sqrt{3}}{\pi \sqrt{20ℵ\xi (\xi +1)}}<Pr\left[complete\right]<\frac{\sqrt{3}}{\pi \sqrt{2ℵ\xi (\xi +1)}}$.*

**Proof.**  First, we evaluate the probability $Pr[1+\sum_{i=1}^{ℵ}{\left(-1\right)}^{\nu_{i}}p_{i}=0|\nu_{i}\overset{\$}{\leftarrow }\left\{0,1\right\},p_{i}\overset{\$}{\leftarrow }\left\{-\xi ,-\xi +1,\cdots ,\xi \right\}]$ since ${\left(-1\right)}^{\nu_{i}}p_{i}$ submits the uniform distribution over {−ξ,−ξ+1,⋯,ξ}, $Pr[{\left(-1\right)}^{\nu_{i}}p_{i}$ = $t|\nu_{i}\overset{\$}{\leftarrow }\left\{0,1\right\},p_{i}\overset{\$}{\leftarrow }\left\{-\xi ,-\xi +1,\cdots ,\xi \right\}$, t∈{−ξ,−ξ+1,⋯,ξ}] = 1/(2ξ+1). The mean and variance are $E[{\left(-1\right)}^{\nu_{i}}p_{i}]=0,$$D\left[{\left(-1\right)}^{\nu_{i}}p_{i}\right]=\frac{1}{2\xi +1}\sum_{i=-\xi }^{\xi }i^{2}=\frac{1}{3}\xi \left(\xi +1\right)$, respectively. Then, $E[\frac{1}{ℵ}\sum_{i=1}^{ℵ}{\left(-1\right)}^{\nu_{i}}p_{i}]=0$, $D\left[\frac{1}{ℵ}\sum_{i=1}^{ℵ}{\left(-1\right)}^{\nu_{i}}p_{i}\right]=\frac{1}{3ℵ}\xi \left(\xi +1\right)$. When ℵ becomes big enough, $\frac{1}{ℵ}\sum_{i=1}^{ℵ}{\left(-1\right)}^{\nu_{i}}p_{i}$ obeys the Gaussian distribution $\psi_{0,\sqrt{\frac{1}{3ℵ}\xi \left(\xi +1\right)}}$, according to the Central Limit Theorem. Then,

(16) $$\begin{array}{c} \begin{array}{cc} \; & Pr[1+\sum_{i=1}^{ℵ}{\left(-1\right)}^{\nu_{i}}p_{i}=0|\nu_{i}\overset{\$}{\leftarrow }\left\{0,1\right\},p_{i}\overset{\$}{\leftarrow }\left\{-\xi ,-\xi +1,\cdots ,\xi \right\}]\\ = & Pr[\frac{1}{ℵ}\sum_{i=1}^{ℵ}{\left(-1\right)}^{\nu_{i}}p_{i}=-\frac{1}{ℵ}|\nu_{i}\overset{\$}{\leftarrow }\left\{0,1\right\},p_{i}\overset{\$}{\leftarrow }\left\{-\xi ,-\xi +1,\cdots ,\xi \right\}]\\ = & \frac{1}{\sqrt{2\pi }\sqrt{\frac{ℵ}{3}\xi \left(\xi +1\right)}}e^{-1/(\frac{2}{3}{ℵ}^{3}\xi \left(\xi +1\right))}\approx \frac{\sqrt{3}}{\sqrt{2\pi ℵ\xi (\xi +1)}} \end{array} \end{array}$$

This probability is non-negligible.

Next, we evaluate the probability that the simulation normally completes the signature queries and forgery with the affine subspaces method, as in [34]. Since $f_{i}=h_{s}p_{i}+e_{i}+p_{i}^{\prime }h_{f}$, $p_{i},e_{i}\overset{\$}{\leftarrow }{\left\{-1,0,1\right\}}^{n}$, $p_{i}^{\prime }\overset{\$}{\leftarrow }\left\{-\xi ,-\xi +1,\cdots ,\xi \right\}$ from i=0 to ℵ except $p_{0}^{\prime }=1$. At the beginning of the game, the values of $p_{i}^{\prime }$s are completely hidden from A by the LWE instances $h_{s}p_{i}+e_{i}$. Even though A knows the calculation form of $f_{i}$, some information is leaked due to signature queries. A can not infer enough information by Bayesian updating to observably increase the probability of forging. For $\nu^{*}$, the event $1+\sum_{i=1}^{ℵ}{\left(-1\right)}^{\nu_{i}^{*}}p_{i}^{\prime }=0$ corresponds a hyperplane $V^{*}$ with $|V^{*}|=\frac{\sqrt{3}}{\sqrt{2\pi ℵ\xi (\xi +1)}}\left|V\right|$, where |V| denotes the size of the total space determined by $\left(p_{1},p_{2},\cdots p_{ℵ}\right)$. Let $V_{j}$ denote a hyperplane that can lead to an abort in the *j*th signature query. Then, $|V^{*}\setminus V_{j}\left|=\right|V^{*}-V^{*}\cap V_{j}|=\frac{\sqrt{3}}{\sqrt{2\pi ℵ\xi (\xi +1)}}\left(1-\frac{\sqrt{3}}{\sqrt{2\pi ℵ\xi (\xi +1)}}\right)\left|V\right|$. The lower bound can be computed by the union bound,

(17) $$\begin{array}{c} \begin{array}{c} Pr\left[complete\right]=Pr\left[\left(p_{1},p_{2}\cdots ,p_{ℵ}\right)\in \left(V^{*}\setminus \cup_{j=1}^{Q}V_{j}\right)\right]\geq \frac{\sqrt{3}}{\sqrt{2\pi ℵ\xi (\xi +1)}}\left(1-\frac{\sqrt{3}Q}{\sqrt{2\pi ℵ\xi (\xi +1)}}\right) \end{array} \end{array}$$

The upper bound can be evaluated trivially, $Pr\left[complete\right]\leq \frac{\sqrt{3}}{\sqrt{2\pi ℵ\xi (\xi +1)}}$.

Specifically, when

$$Q<\frac{9\sqrt{2\pi ℵ\xi (\xi +1)}}{10\sqrt{3}}<\frac{9\sqrt{2\pi ℵ\varrho n(\varrho n+1)}}{10\sqrt{3}}\approx \frac{9\varrho n\sqrt{2\pi ℵ}}{10\sqrt{3}}\approx \frac{\sqrt{3}\pi^{7/2}q}{20n^{2}{ℵ}^{1/2}{\left(\ln\left(2^{(7+\lambda )/2}n\right)\right)}^{3/2}},$$

$\frac{\sqrt{3}Q}{\sqrt{2\pi ℵ\xi (\xi +1)}}<\frac{9}{10}$, $\frac{\sqrt{3}}{10\sqrt{2\pi ℵ\xi (\xi +1)}}<Pr\left[complete\right]<\frac{\sqrt{3}}{\pi \sqrt{2ℵ\xi (\xi +1)}}$.    □

**Theorem** **2.** *If there exists an adversary A who can forge an existential signature for SC-NTRU with probability ϵ by carrying out adaptive chosen-message queries, then a probabilistic algorithm C can be constructed to solve the search LWE${}_{n,m,q}$ problem with parameter settings in Equation (1) in almost the same time with probability $\frac{\sqrt{3}\epsilon }{\pi \sqrt{2ℵ\xi (\xi +1)}}\left(1-\frac{\sqrt{3}Q}{\pi \sqrt{2ℵ\xi (\xi +1)}}\right)$.*

**Proof.**  **Setup**. The challenger C queries the RLWE oracle to fetch a random RLWE(q,n,m,β) instance $\left(h_{s},y\right)$. Then, C generates the simulation environment for A as follows.

1. C generates a public and private key pair by running the KeyGen algorithm $\left(B_{f},h_{f}\right)\leftarrow \mathrm{KeyGen}\left(n,q\right)$.
2. C samples $p_{i},e_{i}\overset{\$}{\leftarrow }{\left\{-1,0,1\right\}}^{n}$, $p_{i}^{\prime }\overset{\$}{\leftarrow }\left\{-\xi ,-\xi +1,\cdots ,\xi \right\}$ from i=1 to ℵ but sets $p_{i}^{\prime }=1$, then computes $f_{i}=h_{s}p_{i}+e_{i}+p_{i}^{\prime }h_{f}$ for *i* from 1 to ℵ.

C returns $\left(h_{f},{\left\{f_{i}\right\}}_{i=0}^{ℵ}\right)$ to A.

**Queries**. A submits a randomly chosen message $\mu \in {\left\{0,1\right\}}^{*}$ to C to query. C replies to the query by generating the corresponding signature as follows.

1. $k\overset{\$}{\leftarrow }{\left\{0,1\right\}}^{\lambda }$.
2. ν: $=H_{0}\left(\mu ,b_{r},k\right)$.
3. $p^{\prime }=p_{0}^{\prime }+\sum_{i=1}^{ℵ}{\left(-1\right)}^{\nu_{i}}p_{i}^{\prime }$. If $p^{\prime }=0$, go to step 1.
4. $p=p_{0}+\sum_{i=1}^{ℵ}{\left(-1\right)}^{\nu_{i}}p_{i}$.
5. f: $=f_{0}+\sum_{i=1}^{ℵ}{\left(-1\right)}^{\nu_{i}}f_{i}$.
6. e: $=e_{0}+\sum_{i=1}^{ℵ}{\left(-1\right)}^{\nu_{i}}e_{i}$.
7. Generate a basis B:= SimExtractS $\left(h_{s},p,e,p^{\prime },h_{f},B_{f}\right)$ for $\left(h_{s},f\right)$ (see Algorithm 2).
8. $\left(x^{\prime },x\right)$: =(y,0)−SamplePre(B,η,(y,0)).
9. return x as the signature.

**Forgery**. C receives a forgery x signature on a new message μ, then constructs the solution for ISIS as follows.

1. Compute $p^{\prime }=p_{0}^{\prime }+\sum_{i=1}^{ℵ}{\left(-1\right)}^{\nu_{i}}p_{i}^{\prime }$. If $p^{\prime }\neq 0$, abort.
2. $p=p_{0}+\sum_{i=1}^{ℵ}{\left(-1\right)}^{\nu_{i}}p_{i}$.
3. Divide x as $\left(x_{0},x_{1}\right)\in Z_{q}^{n}\times Z_{q}^{n}$.
4. Return $\left(x_{0}+px_{1},y-h_{s}\left(x_{0}+px_{1}\right)\right)$ as the solution for $\left(h_{s},y\right)$.

Firstly, according to Lemma 16, the simulated signatures generated during the query phase are valid and indistinguishable from the real signatures. That is, C creates a simulated environment, indistinguishable from reality for adversaries. This will lead A to complete the reduction game.

Secondly, according to Lemma 17, the coefficient vector of $y-h_{s}\left(x_{0}+px_{1}\right)$ is short enough under the parameter settings in Equation (1). Therefore, the polynomials generated in step 4 of the forgery phase form a solution for the RLWE instance $\left(h_{s},y\right)$.

Finally, according to Lemma 18, the simulation can successfully complete both signature queries and forgery without aborting, with non-negligible probability.

In summary, C can obtain a solution for an RLWE instance with non-negligible probability, if A has the ability to forge a valid signature for the SC-NTRU scheme.    □

**Algorithm 2** SimExtractS$(h_{s},p,e,p,h_{f},B_{f}$)**Require:** public keys $h_{s},h_{f}\in R_{q}$ and syndrome $u\in R_{q}$, in which $h_{f}\leftarrow KeyGen$, $b_{r}=h_{r}p+e$ for $p,e\leftarrow {\left\{-1,0,1\right\}}^{n}$; a pair of un-equivalent tags $\tau ,\tau^{*}$; a syndrome $u\in R_{q}$; a basis $B_{f}$ for $h_{f}$.**Ensure:**  A private key $\left(x_{1},x_{2}\right)$.
 1:$f=h_{s}p+e+ph_{f}$ 2:Γ={} 3:**repeat** 4: $r\overset{\$}{\leftarrow }\left\{0,1\right\}$ 5: **if** r=0 **then** 6:  $x_{0}^{\prime }\leftarrow D_{Z^{n},\eta_{0},0}$ (where $\eta_{0}=\frac{1}{\pi }\sqrt{\frac{1}{2}\ln\left(2^{(7+\lambda )/2}n\right)}$ ) 7:  $y^{\prime }=\left(-h_{s}x_{0}^{\prime }\right)/p$ 8:  $\left(x_{1}^{\prime },x_{1}\right)$: $=\left(y^{\prime },0\right)-\mathrm{SamplePre}\left(B_{f},\eta_{3},\left(y^{\prime },0\right)\right)$ where $\eta_{3}=\frac{1.17}{\pi }\sqrt{\frac{1}{2}\ln\left(2^{(7+\lambda )/2}n\right)q}$ 9:  $x_{0}=x_{0}^{\prime }-px_{1}$ 10: **else** 11:  $\left(x_{1}^{\prime },x_{1}\right)$: $=(g_{f_{i}},t_{f_{i}})$ where $\left(g_{f_{i}},t_{f_{i}}\right)$ is a basis vector of $\Lambda_{f,q}$ 12:  $x_{0}=-px_{1}$ 13: **end if** 14: $b=(x_{0},x_{1})$ 15: **if** b is linearly independent with the vectors in Γ **then** 16:  Γ=Γ∩b 17: **end if** 18:**until**|Γ|=2n 19:covert 2n linearly independent vectors in Γ to a basis B of $\left(h_{s}∥f\right)$ with the algorithm in Lemma 7.1 of [44].

### 5.2. Performance

In the existing lattice-based signcryption schemes, some building blocks are often used, such as the algorithm SampleD of [18], Inver of [18], SamplePre of [41], inverting matrix and solving system of linear equations, etc. To facilitate the performance comparison, we summarize some basic conclusions about the computational cost for these building blocks. In order to clearly state these conclusions, we introduce some notations for the basic mathematical operations. Let $\times_{Z}$, $\times_{Z_{q}}$, $\times_{R}$ denote the multiplication operation over Z, $Z_{q}$, R, respectively. Let ${+}_{Z}$, ${+}_{Z_{q}}$, ${+}_{R}$ denote the addition operation over Z, $Z_{q}$, R, respectively.

The computational overhead of matrix inversion and solving systems of linear equations can be evaluated by regular computation.

**Proposition** **5.** *The computational cost to invert an n-dimensional matrix over $Z_{q}$ is about $\frac{2}{3}n\left(n^{2}+3n-1\right)$ multiplications over $Z_{q}$.*

**Proposition** **6.** *The computational cost of solving n×m-dimension nonhomogeneous linear equations over $Z_{q}$ is about $\frac{1}{2}n\left(n+1\right)\left(m+1\right)+\frac{1}{6}\left(n-2\right)\left(n-1\right)\left(n+3\right)$ multiplications and additions over $Z_{q}$, and 2nO(logq) inversion over $Z_{q}$. Here, the equation substitution is $mn-\frac{1}{2}n\left(n-1\right)$ multiplications and $mn+\frac{1}{2}n\left(n+1\right)$ additions over $Z_{q}$ and O(logq) inversion over $Z_{q}$.*

Micciancio and Peikert presented a pre-image sample algorithm named SampleD${}^{O}$, specifically designed for the MP trapdoor [18]. For more details, please refer to [18]. The computational overhead of the algorithm can be broken down into the following steps:

- Step 1: Generating (2nlogq)-dimension DGS.
- Step 2: Performing $\left(n^{2}\log^{2}q+n^{2}\log q\right)$$\times_{Z}$ and ${+}_{Z}$, as well as $\left(n^{2}\log q\right)$$\times_{Z_{q}}$ and ${+}_{Z_{q}}$.
- Step 3: Conducting $\left(n^{2}\right)$$\times_{Z}$ and ${+}_{Z}$, $\left(n^{2}\right)$$\times_{Z_{q}}$ and ${+}_{Z_{q}}$, and utilizing nlogq-dimension DGS.
- Step 4: Involving $\left(n^{2}\log^{2}q\right)$$\times_{Z}$ and ${+}_{Z}$.

Therefore, the overall computational overhead of SampleD${}^{O}$ is summarized as follows.

**Proposition** **7.** *The computational cost of Algorithm SampleD${}^{O}$ of [18] is about 3nlogq discrete Gaussian samples (DGS), $2n^{2}\log^{2}q+n^{2}\log q+n\log q$ multiplications and additions over Z, $n^{2}\log q+n^{2}$ multiplications and additions over $Z_{q}$. The overhead to compute the Gaussian parameter is $3n^{3}\log^{3}q$, which can be precomputed.*

Except for SampleD, the computational cost of signcryption of [17] is about 5nlogq DGS, $8n^{2}\log q$ multiplications over $Z_{q}$, $\left(\lambda +8\right)n^{2}\log q+n\log q-n$ additions over $Z_{q}$, $n^{2}\log^{2}q+n\log q$ multiplications over Z, $n^{2}\log^{2}q-n\log q$ additions over Z.

In [18], an inversion algorithm called Inver is presented for the MP trapdoor. The computational overhead of the steps is described as follows.

- Step 1: Involves $\left(n^{2}\log^{2}q\right)$ $\times_{Z}$ and ${+}_{Z}$.
- Step 2: Includes (nlogq) $\times_{Z}$ and ${+}_{Z}$, as well as (nlogq) $\times_{Z_{q}}$ and ${+}_{Z_{q}}$.
- Step 3: Requires $\left(2n^{2}\log q+n^{2}\right)$ $\times_{Z_{q}}$ and ${+}_{Z}$.

Therefore, the total computational overhead of Algorithm Inver is as follows.

**Proposition** **8.** *The computational cost of Algorithm **Inver** of [18] is about $n^{2}\log^{2}q+2n^{2}\log q+n^{2}+n\log q$ multiplications and $n^{2}\log^{2}q+2n^{2}\log q+n^{2}-2n$ additions over $Z_{q}$, 2nlogq multiplications and additions over Z. In the process, the cost of the inversion oracle is 2nlogq multiplications and additions over Z.*

In addition to solving system of linear equations, Algorithm SamplePre also involves executing the randomized nearest-plane algorithm. The computational cost of its steps is as follows.

- step a: Involves (2m) $\times_{R}$ and (2m−2) ${+}_{R}$.
- step b: Requires 1 DGS.
- step c: Needs *m* $\times_{Z}$ and (2m) ${+}_{Z}$.

The procedure is carried out *m* times. Consequently, the total computational cost of SamplePre without solving equations is calculated as follows.

**Proposition** **9.** *Except for solving equations, the computational cost of Algorithm SamplePre of [41] is about 2 $m^{2}$ multiplications and additions over R, $m^{2}$ multiplications and 2 $m^{2}$ additions over Z and m DGS.*

The operations involved in Algorithm Gaussian_Sampler of [38] are almost identical to those in Algorithm SamplePre except for the solving equations part. However, the dimension of the basis used in Gaussian_Sampler is 2n×2n. Consequently, its computational overhead includes $\left(8n^{2}\right)$ $\times_{R}$ and ${+}_{R}$, $\left(4n^{2}\right)$ $\times_{Z}$ and $\left(8n^{2}\right)$ ${+}_{Z}$, and 2n DGS. However, the circulant property of the basis matrix allows for the use of fast Fourier orthogonalization, which can speed up the procedure significantly [45]. According to [45], when adopting fast Fourier orthogonalization, the complexity of the Gaussian_Sampler is given as follows.

**Proposition** **10.** *The computational cost of Algorithm Gaussian_Sampler of [38] is about 2n DGS, 4Θ(nlogn) multiplications and additions over R, 4Θ(nlogn) multiplications and additions over Z.*

In Table 3, the sizes of public parameters, public key, private key, ciphertext, and security are compared. The sizes of the public parameters and the public key of SC-NTRU are approximately in the order of magnitude of 1/(nlogq) of those of YWW+13 [17], SS18 [19], and YCL+19 [20]. The private key size of SC-NTRU is roughly in the order of magnitude of $2/(n\log^{2}q)$ of those of YWW+13 [17] and SS18 [19], and $4/(n\log^{2}q)$ of that of YCL+19 [20]. Except for the plaintext length, the ciphertext size of SC-NTRU is about at the order of magnitude of 1/logq of those of YWW+13 [17], SS18 [19], and YCL+19 [20]. Regarding security, the proposed scheme achieves IND-CCA2 security against adaptively chosen ciphertext attacks, similar to the other schemes in the table. However, when facing adaptively chosen message attacks, the proposed scheme is EUF-CMA secure instead of SUF-CMA security, as in YWW+13 [17] and SS18 [19]. Our intention is to demonstrate that a EUF-CMA secure signature component is sufficient to guarantee the IND-CCA2 security of the signcryption ciphertext.

In Table 4, the computational overhead of YWW+13 [17], SS18 [19], YCL+19 [20], and SC-NTRU is compared. First, the cost of the key generation is compared. In the key generation phase of YWW+13 [17] and SS18 [19], the computation of $\left(\frac{2}{3}n^{3}+2n^{2}\right)\;\times_{Z_{q}}$ is required to invert the matrix H. As it is the repeating computation in signcryption, it is reasonable to count its overhead into the key generation instead of signcryption. The numbers of $\times_{Z_{q}}$ and ${+}_{Z_{q}}$ of SC-NTRU are in the order of magnitude of 1/n of those of YWW+13 [17] and SS18 [19], and at a lower order of magnitude of YCL+19 [20]. The number of DGS of SC-NTRU is about $2/(n\log^{2}q)$ of those of YWW+13 [17] and SS18 [19]. In SC-NTRU, the samples from $U(Z_{q})$ are not needed; however, the numbers of samples reach $n^{2}\log q$ in YWW+13 [17] and SS18 [19].

Next, the overhead of the signcryption is compared. The $\times_{Z_{q}}$ and ${+}_{Z_{q}}$ operations in SC-NTRU are in the order of magnitude of 1/n of YWW+13 [17] and SS18 [19], respectively. The numbers of $\times_{Z}$ and ${+}_{Z}$ of SC-NTRU are in the order of magnitude of 1/(nlogq) of those of YWW+13 [17] and SS18 [19], and at a lower order of magnitude of YCL+19 [20]. The number of DGS of SC-NTRU is no more than 1/(4logq) of those of YWW+13 [17] and SS18 [19], and at a lower order of magnitude of YCL+19 [20]. Compared with YWW+13 [17] and SS18 [19], SC-NTRU requires (4θ(nlogn)) additional $\times_{R}$ and ${+}_{R}$, and they are about $4/(n\log^{3}q)$ of YCL+19 [20]. In summary, although the schemes of YWW+13 [17] and SS18 [19] are built on the ordinary lattice, their signcryption performance significantly outperforms that of YCL+19 [20], due to not requiring the expensive basis extension. However, their signcryption cost is several orders of magnitude higher than that of SC-NTRU.

Finally, the cost of unsigncryption is compared. The numbers of $\times_{Z_{q}}$ and ${+}_{Z_{q}}$ operations of SC-NTRU are in the order of magnitude of 1/n and 1/(nlogq+ℓ) of those of YWW+13 [17] and SS18 [19], respectively. The numbers of $\times_{Z}$ and ${+}_{Z}$ operations of SC-NTRU are in the order of magnitude of 1/(nlogq) and 1/(nlogqℓ) of those of YWW+13 [17] and SS18 [19], respectively. Compared with YWW+13 [17], SC-NTRU needs 3n additional DGS; however, it is only 1/(kℓ) of that of SS18 [19]. The numbers of DGS, $\times_{Z_{q}}$ and ${+}_{Z_{q}}$, $\times_{Z}$ and ${+}_{Z}$, $\times_{R}$ and ${+}_{R}$ of SC-NTRU are in the order of magnitude of 3/(2logq), 1/logq, 4/logq, and 4/logq of those of YCL+19 [20]. In summary, since YCL+19 [20] adopts the ideal lattice, the performance of the unsigncryption greatly outperforms YWW+13 [17] and SS18 [19]; however, its overheads are logq/4 to 2logq/3 of those of SC-NTRU.

To sum up, due to the efficiency of the NTRU trapdoor and reasonable construction, the proposed scheme achieves orders of magnitude of improvement in computation cost over the existing signcryption schemes.

### 5.3. Experiment

To assess the actual performance of SC-NTRU, we conducted experiments using the C programming language. The experimental environment is set up on a Ubuntu platform with 4 GB of memory. The dimension of the NTRU lattice has a significant impact on security. Therefore, we choose three sets of typical parameters: n = 256, 512, and 1024, corresponding to low, medium, and high levels of security, respectively. We run the experiment 1000 times and calculate the average time. The experimental data are presented in Table 5. According to the data in the table, the running time of key generation, signcrypt, and unsigncrypt exhibit running times on the order of milliseconds. Notably, the running time of signcrypt (and unsigncrypt) ranges between 1.3 and 6.2 (1.1 and 5.5), demonstrating the efficiency of SC-NTRU.

## 6. Conclusions

In this paper, a signcryption scheme following the StE paradigm is proposed based on the intractability of the NTRU lattice and RLWE, which serves as the security foundation of Falcon in NIST PQC. First, it is shown how to embed some sensitive information into a general lattice-based public key encryption (PKE) and bind it with the message being encrypted by PKE. The malleability to the ciphertext ultimately leads to the modification in the message–signature pair. Consequently, the signature for the message can also be utilized to verify and guarantee the IND-CCA2 security of the ciphertext. Thus, the need for the MAC to transfer from the public key to the signature is eliminated.

Secondly, a new abort-resistant hash is proposed to match the “partiality” of the pre-image in relation to the checkout polynomial, so that an NTRU signature secure in the standard model can be built with it. The computational overhead analysis demonstrates a significant improvement in the efficiency of SC-NTRU, surpassing existing lattice-based signcryption methods by orders of magnitude. The experiment shows that SC-NTRU is very efficient. 

## Figures and Tables

**Table 1:** **Game IND-CCA2** between C and A.

	C	Communication	A
Intial.	$\left\{\left(pk_{s},sk_{s}\right),\left(pk_{r},sk_{r}\right)\right\}\leftarrow \mathrm{KeyGen}\left(1^{k}\right)$	$\overset{pk_{s},pk_{r},sk_{s}}{\to }$	
Phase 1.		$\overset{c}{\underset{\mathrm{for}\;\;\mathrm{Unsigncrypt}}{\leftarrow }}$	choose *c*
	$\mu =\mathrm{UnSigncrypt}(c,pk_{r},pk_{s},sk_{r})$	$\overset{\mu }{\underset{\mathrm{as}\;\;\mathrm{reply}}{\to }}$	
	…	…	…
Challenge		$\overset{\mu_{0},\mu_{1}}{\underset{\mathrm{for}\;\;\mathrm{challenge}}{\leftarrow }}$	choose $\mu_{0},\mu_{1}$ with equal length
	Toss a coin *b*, $c^{*}=\mathrm{Signcrypt}\left(\mu_{b},pk_{r},pk_{s},sk_{s}\right)$	$\overset{c^{*}}{\underset{\mathrm{as}\;\;\mathrm{reply}}{\to }}$	
Phase 2.	repeat Phase 1, except reply ⊥ to the query for $c^{*}$	repeat	repeat
Guess		$\overset{b^{\prime }}{\underset{\mathrm{as}\;\;\mathrm{reply}}{\leftarrow }}$	guess $b^{\prime }$

**Table 2:** **Game EUF-CMA** between C and A.

	C	Communication	A
Initial.	$\left\{\left(pk_{s},sk_{s}\right),\left(pk_{r},sk_{r}\right)\right\}\leftarrow \mathrm{KeyGen}\left(1^{k}\right)$	$\overset{pk_{s},pk_{r},sk_{r}}{\to }$	
Queries		$\overset{\mu }{\underset{\mathrm{for}\;\;\mathrm{Signcrypt}}{\leftarrow }}$	choose μ
	$c=\mathrm{SC}(\mu ,pk_{r},pk_{s},sk_{s})$	$\overset{c}{\underset{\mathrm{as}\;\;\mathrm{reply}}{\to }}$	
	…	…	…
Forgery		$\overset{c^{*}}{\underset{\mathrm{as}\;\;\mathrm{reply}}{\leftarrow }}$	generate $c^{*}$

**Table 3:** Comparison of the key size, public parameter size, and security of the related schemes.

Scheme	YWW+13 [17]	SS18 [19]	YCL+19 [20]	Ours
public parameter	$\left(\lambda +3\right)n^{2}\log^{2}q$ +nℓlogq	$\left(\lambda +3\right)n^{2}\log^{2}q$ +nℓlogq	$\left(2k^{\prime }+\ell +5\right)$ $n^{2}\log^{2}q$	(ℵ+1)nlogq ^1^
public key	$3n^{2}\log^{2}q$ +nℓlogq	$3n^{2}\log^{2}q$ +nℓlogq	$n^{2}\log^{2}q$	3nlogq
private key	$24n^{2}\log^{2}q\log\sigma$ ^2^	$24n^{2}\log^{2}q\log\sigma$	$12n^{2}\log^{2}q\log\sigma$	48nlogσ
ciphertext	$2n\log^{2}q+\left|\mu \right|$ $+48n\log q\log\sigma_{1}$ ^3^	$2n\log^{2}q+\left|\mu \right|$ +12(7n+ℓ)logq $\cdot \log\sigma_{2}$	$4n\log^{2}q+\left|\mu \right|$ $+36n\log q\log\sigma_{3}$ +n+ℓ	3nlogq+|μ| $+24n\log\sigma_{4}$
security	IND-CCA2, SUF-CMA	IND-CCA2, SUF-CMA	IND-CCA2, EUF-CMA	IND-CCA2, EUF-CMA
based on NIST	No	No	No	Yes

^1^ λ, ℓ, ℵ are all with the security parameter, so they are roughly the same. ^2^ Every element of the private key is stored in 12logσ, when its Gaussian parameter is σ. ^3^ |μ| denotes the length of the message.

**Table 4:** Comparison of the computation overhead of the related signcryption schemes.

	Computation Type	YWW+13 [17]	SS18 [19]	YCL+19 [20]	Ours
Setup	$\leftarrow U(Z_{q})$	$\left(\lambda +3\right)n^{2}\log q$ +nℓ	$\left(\lambda +3\right)n^{2}\log q$ +nℓ+n	(ℓ+5)nlogq	(ℵ+5)n
KeyGen	$\leftarrow U(Z_{q})$	$n^{2}\log q$	$n^{2}\log q$	σn((m−2σ−r) /⌈logq⌉+r+2)	0
	DGS	$n^{2}\log^{2}q$	$n^{2}\log^{2}q$	0	2n
	$\times_{Z_{q}}$	$n^{3}\log^{2}q$ $+\frac{2}{3}n^{3}+2n^{2}$	$n^{3}\log^{2}q$ $+\frac{2}{3}n^{3}+2n^{2}$	(m−2σ−r) (r+σ)	$7O(n^{2}\log n)$ +8O(nlogn)
	${+}_{Z_{q}}$	$n^{3}\log^{2}q$ $+\frac{2}{3}n^{3}+2n^{2}$	$n^{3}\log^{2}q$ $+\frac{2}{3}n^{3}+2n^{2}$	(m−2σ−r) (r+σ)	$7O(n^{2}\log n)$ +8O(nlogn)
	$\times_{Z}$	0	0	0	4n
	${+}_{Z}$	0	0	0	4n
Signcrypt	DGS	8nlogq	9nlogq+ℓ	$n^{2}\log^{2}q$ +6nlogq+n	2n
	$\times_{Z_{q}}$	$10n^{2}\log q$ +2nlogq $+n^{2}+n\ell$	$10n^{2}\log q$ +nlogq $+n^{2}+n\ell$	nlogq(3logq+8) ·O(nlogn)+ $\left(2\log q-1\right)n^{2}\log q$	5O(nlogn)
	${+}_{Z_{q}}$	$\left(\lambda +10\right)n^{2}\log q$ $+n^{2}+n\ell -2n$	$\left(\lambda +11\right)n^{2}\log q$ $+n^{2}+n\ell -5n$ +4nlogq+ℓ	nlogq(3logq+8) ·O(nlogn)+nℓ+ $\left(2\log q-1\right)n^{2}\log q$	5O(nlogn) +(ℵ+3)n
	$\times_{Z}$	$2n^{2}\log^{2}q$ $+n^{2}\log q$ $+3n\log q+n^{2}$	$2n^{2}\log^{2}q$ $+n^{2}\log q$ +3nlogq+ℓ	$\left(n\log^{3}q+2\log^{2}q\right)$ ·O(nlogn)	4θ(nlogn)
	${+}_{Z}$	$2n^{2}\log^{2}q$ $+n^{2}\log q$ $+2n\log q+n^{2}$	$2n^{2}\log^{2}q$ $+n^{2}\log q$ +nlogq	$\left(n\log^{3}q+2\log^{2}q\right)$ ·O(nlogn)+nlogqℓ	4θ(nlogn)
	$\times_{R}$	0	0	$\left(n\log^{3}q+2\log^{2}q\right)$ ·O(nlogn)	4θ(nlogn)
	${+}_{R}$	0	0	$\left(n\log^{3}q+2\log^{2}q\right)$ ·O(nlogn)	4θ(nlogn)
UnSigncrypt	DGS	0	3nkℓ	2nlogq	3n
	$\times_{Z_{q}}$	$8n^{2}\log q$ +nℓ	$n^{2}\log^{2}q$ $+n^{2}\log q\left(\ell +11\right)$ $+n^{2}\left(\ell +1\right)$ +2nlogqℓ	(8logq+1)O(nlogn) +O(nlogqlog(nlogq))	4O(nlogn) +O(2nlog2n)
	${+}_{Z_{q}}$	$8n^{2}\log q$ +λnlogq +nℓ−n	$n^{2}\log^{2}q+n^{2}$ logq(λ+ℓ+11) $+n^{2}\left(\ell +1\right)$ +2nlogq(2ℓ+1)	(8logq+1)O(nlogn) +O(nlogqlog(nlogq)) +6nlogq	4O(nlogn) +O(2nlog2n) +ℵn
	$\times_{Z}$	$2n^{2}\log^{2}q$ +8nlogq +2logq	$2n^{2}\log^{2}q\ell$ $+n^{2}\log q\ell$ +2nlogqℓ +5nlogq+2ℓ	logqO(nlogn) +2nlogq	4θ(nlogn) +5n
	${+}_{Z}$	$2n^{2}\log^{2}q$ +6nlogq +2logq	$2n^{2}\log^{2}q\ell$ $+n^{2}\log q\ell$ +2nlogqℓ +5nlogq+2ℓ	logqO(nlogn) +2nlogq	4θ(nlogn) +3n
	$\times_{R}$	0	0	logqO(nlogn) +2nlogq	4θ(nlogn)
	${+}_{R}$	0	0	logqO(nlogn) +2nlogq	4θ(nlogn)

**Table 5:** Actual performance of SC-NTRU under different parameters.

n	q	Keygen (ms)	SC (μs)	USC (μs)
256	52,145,447,681	16.45	1342.48	1198.03
512	425,478,982,619	36.17	2904.77	2585.58
1024	3,470,791,299,527	95.81	6134.78	5436.07

## Data Availability

Data is contained within the article.
